# Supplementary material for: Somatic mutations in clonally expanded cytotoxic T lymphocytes in patients with newly diagnosed rheumatoid arthritis
Source: Nat Commun. 2017 Jun 21;8:15869. doi: 10.1038/ncomms15869 (PMC5482061; doi:10.1038/ncomms15869)
Supplement: Supplementary Information [file ncomms15869-s1.pdf]

Type of file: pdf
Title of file for HTML: Supplementary Information
Description: Supplementary Figures, Supplementary Tables and Supplementary References

Type of file: xlsx
Title of file for HTML: Supplementary Data 1
Description: The file presents all putative coding single nucleotide variants that were identified in the immunopanel sequencing and in the exome sequencing of RA patients' and healthy controls' lymphocyte fractions.

Type of file: PDF
Title of file for HTML: Peer Review File
Description:

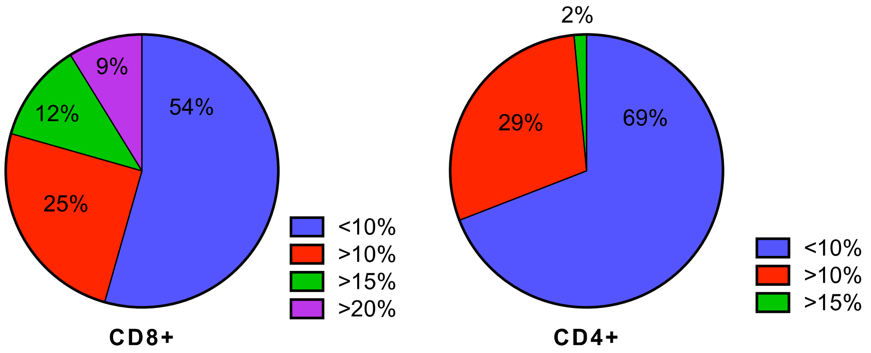

Supplementary Figure 1. Flow cytometry based screening of clonality of CD4+ and CD8+ cells. A panel of monoclonal antibodies designed to cover 70% of the total T-cell pool was used to simultaneously assess the Vβ distribution of both CD4+ and CD8+ T cells in 68 patients. The results show that CD8+ T cells have more populations expressing the same Vβ. Populations comprising over 10% of all CD8+ T cells occurred in 46% of patients, whereas similar populations occurred in CD4+ in 31% of patients. This suggests that CD8+ T cells harbor more clonally expanded cell populations than CD4+ T cells.

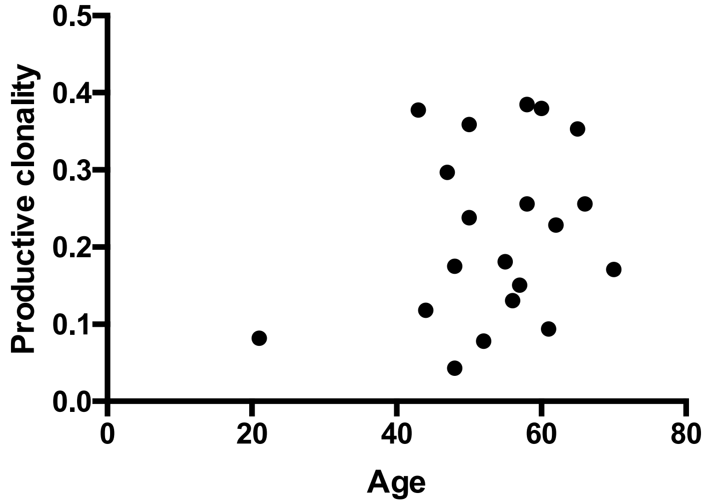

Supplementary Figure 2. CD8+ clonality of 20 healthy controls does not correlate with age. The productive clonality index of CD8+ cells was correlated with the age of the 20 healthy controls included in our study. There was no statistically significant correlation (Spearman correlation coefficient r=0.2183, p=0.3552). However, a correlation was seen in RA patients (Figure 1d). This may be due to the narrower age range and lower sample amount of the healthy controls.

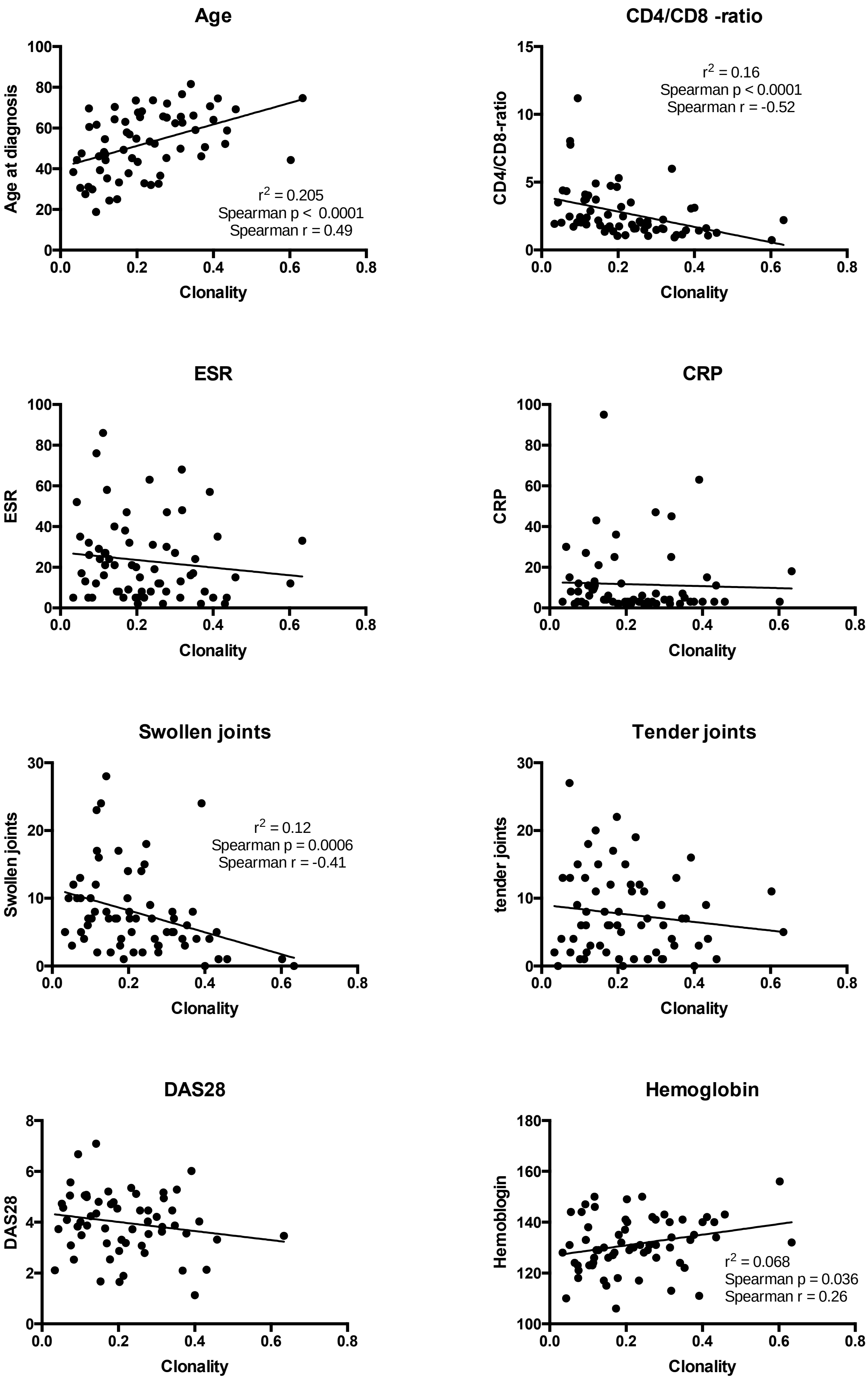
Age
Age at diagnosis
r2 = 0.205
Spearman p < 0.0001
Spearman r = 0.49
Clonality
CD4/CD8 -ratio
CD4/CD8-ratio
r2 = 0.16
Spearman p < 0.0001
Spearman r = -0.52
Clonality
ESR
ESR
Clonality
CRP
CRP
Clonality
Swollen joints
Swollen joints
r2 = 0.12
Spearman p = 0.0006
Spearman r = -0.41
Clonality
Tender joints
tender joints
Clonality
DAS28
DAS28
Clonality
Hemoglobin
Hemoblogin
r2 = 0.068
Spearman p = 0.036
Spearman r = 0.26
Clonality

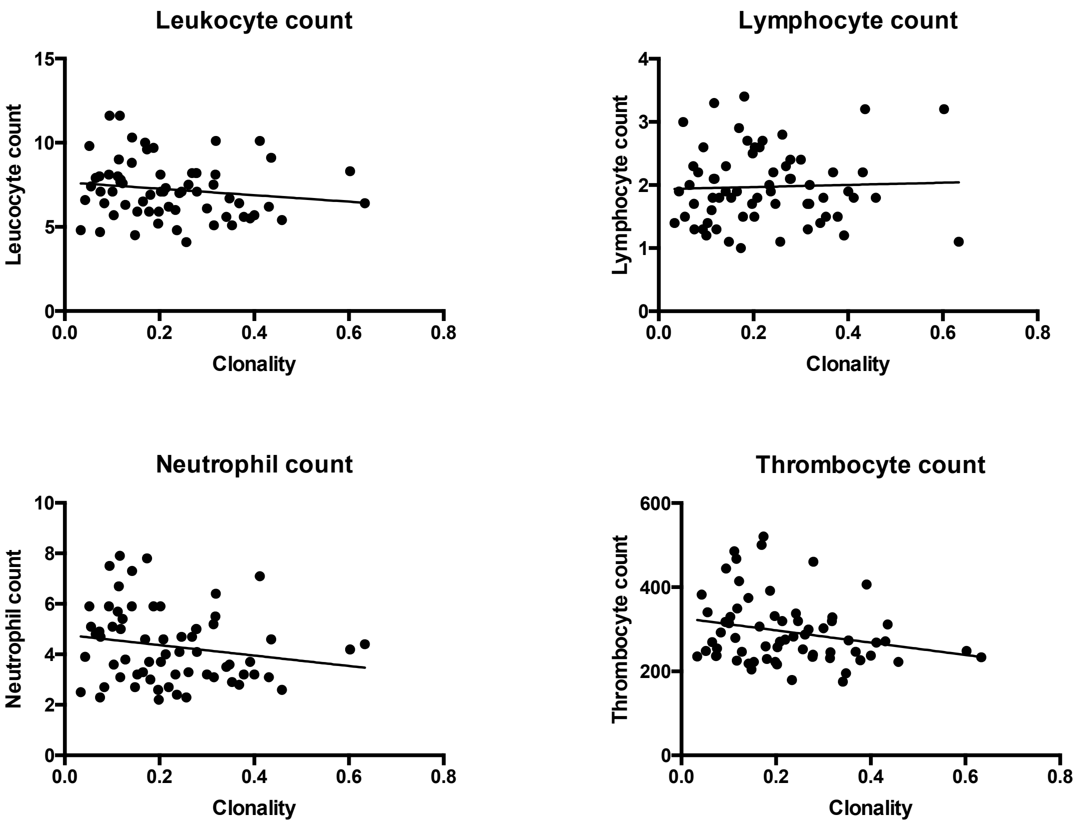

Supplementary Figure 3. Clonality and clinical parameters. Clonality was plotted with clinical variables, and correlations were tested with Spearman correlation. Statistically significant associations are shown in the plots (Linear regression r^2, Spearman correlation coefficient r and p-values). All blood-cell counts are shown as 10^9/l, and in all cases the clonality has been calculated based only on productive clones.

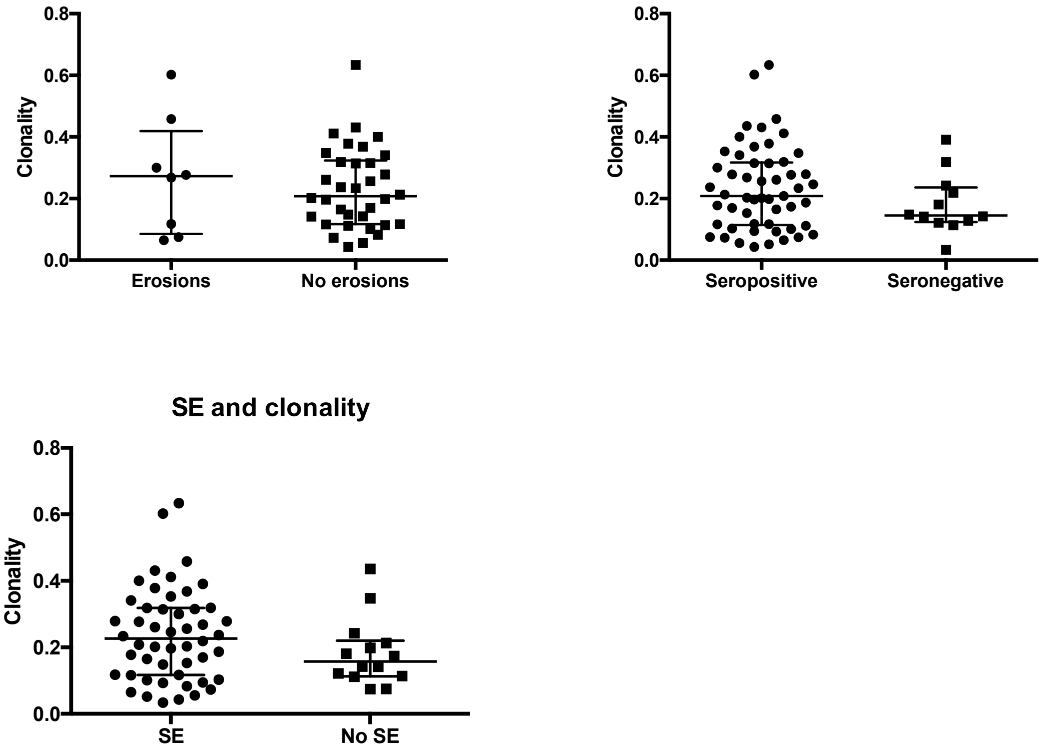

Supplementary Figure 4. Associations of seropositivity, radiographic erosions, and shared epitope alleles with clonality. The CD8 clonality index scores of patients with radiographic erosions in hands or feet at diagnosis were compared to the scores of patients without radiographic erosions. Similarly, the clonality of seropositive and seronegative patients, as well as the clonality of patients with shared epitope (SE)[1] and no shared epitope, was compared. No statistically significant difference emerged between groups (Mann Whitney test). Seropositivity was defined by rheumatoid factor or anti-citrullinated antibody titers larger than >30 IU/ml and U/ml, respectively. These values are considered strongly positive. In all cases, the clonality has been calculated based only on productive clones. Horizontal lines represent group medians and error bars the interquartile range.

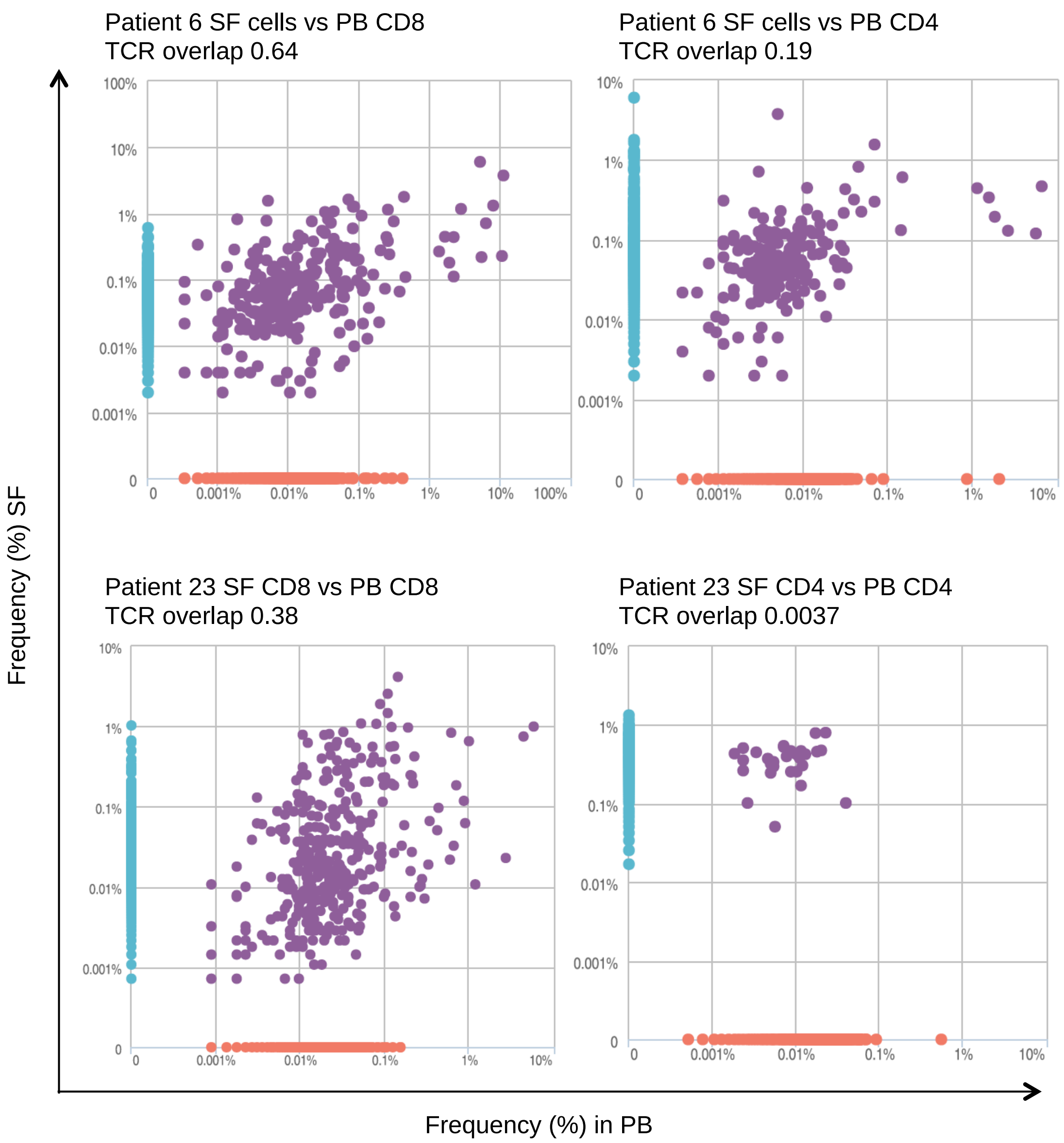

Supplementary Figure 5. The analysis of identical CD8+ clones in peripheral blood and synovial fluid. Same TCRB amino-acid sequences were detected by deep TCRB sequencing in synovial fluid (SF) and peripheral blood (PB) cells. We had access to only two synovial fluid samples from two patients. In the plots, violet dots represent clones (defined by same nucleotide sequence in TCRB sequencing) that were detected in both SF and PB. From patient 6, selected cells from SF were not available, and the sequencing was performed from unselected SF cell DNA. Thus, the percentages are not directly comparable. Patient 6 harbored a clone at 11.1% of all CD8 in peripheral blood (PB), and the same clone comprised 3.8% of all productive synovial fluid (SF) TCRB rearrangements. Another clone occurred in PB at 5.3% and in SF at 6.0%. In patient 23, the largest clone in blood occurred at 5.8% of CD8+ and was 1.0% of SF CD8+ cells. In this patient, sorted cells were available from both peripheral blood and synovial fluid.

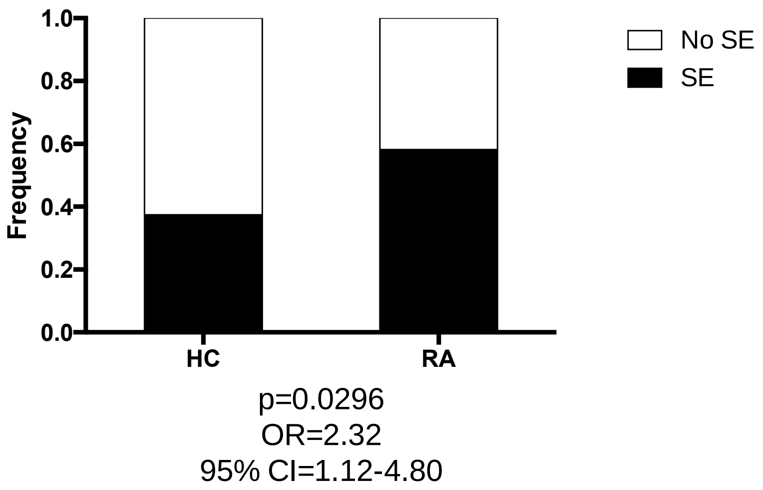

Supplementary Figure 6. Shared epitope frequencies in RA patients and healthy controls. We determined HLA-DRB1 allele frequencies in the study patients to characterize the patient cohort. In RA patients, the frequency of shared epitope alleles (HLA-DRB1*01, HLA-DRB1*04, HLA-DRB1*10, HLA-DRB1*14:02 alleles)[1] of all alleles was 58%, whereas in healthy controls the frequency was 37,5%. The difference between the groups was tested with Fisher's test. Abbreviations: HC, healthy control; RA, rheumatoid arthritis; SE, shared epitope; OR, odds ratio; CI, confidence interval.

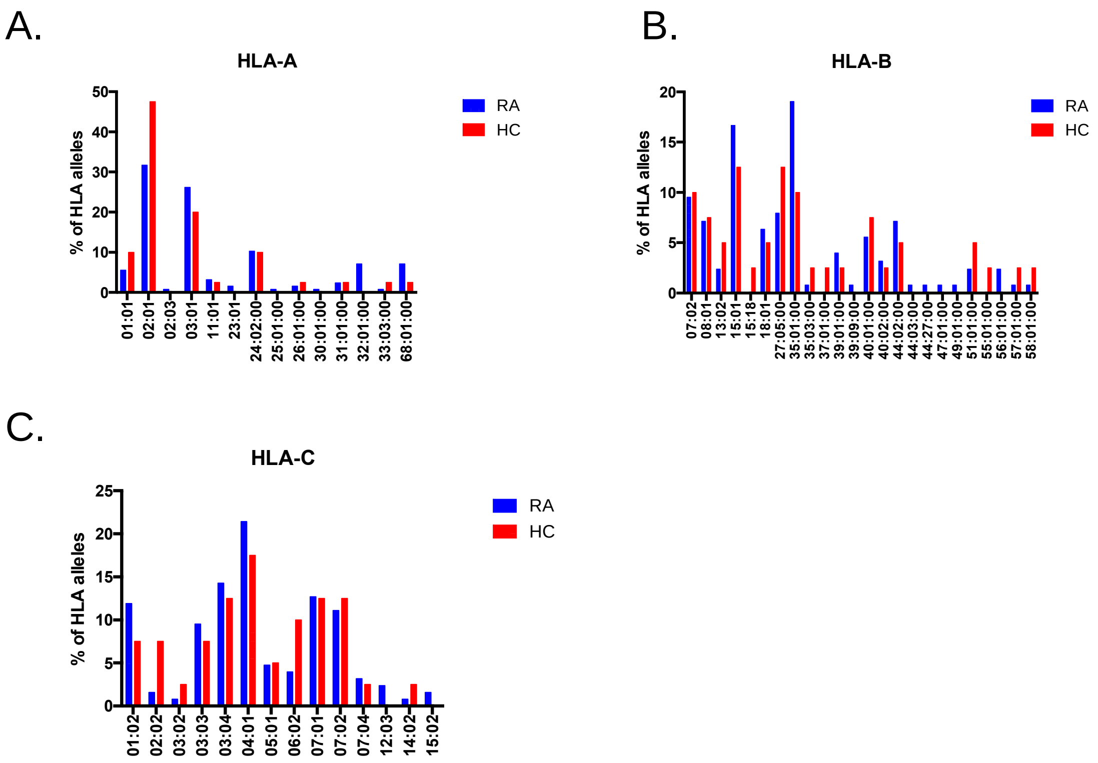

Supplementary Figure 7. HLA-I allele frequencies in patients and healthy controls. The HLA-I alleles of 65 patients and 20 healthy controls were determined, and the frequency of each allele is shown in each plot. There were no statistically significant differences between the patients and healthy controls in any allele frequency (Fisher's test).

A: PADI4

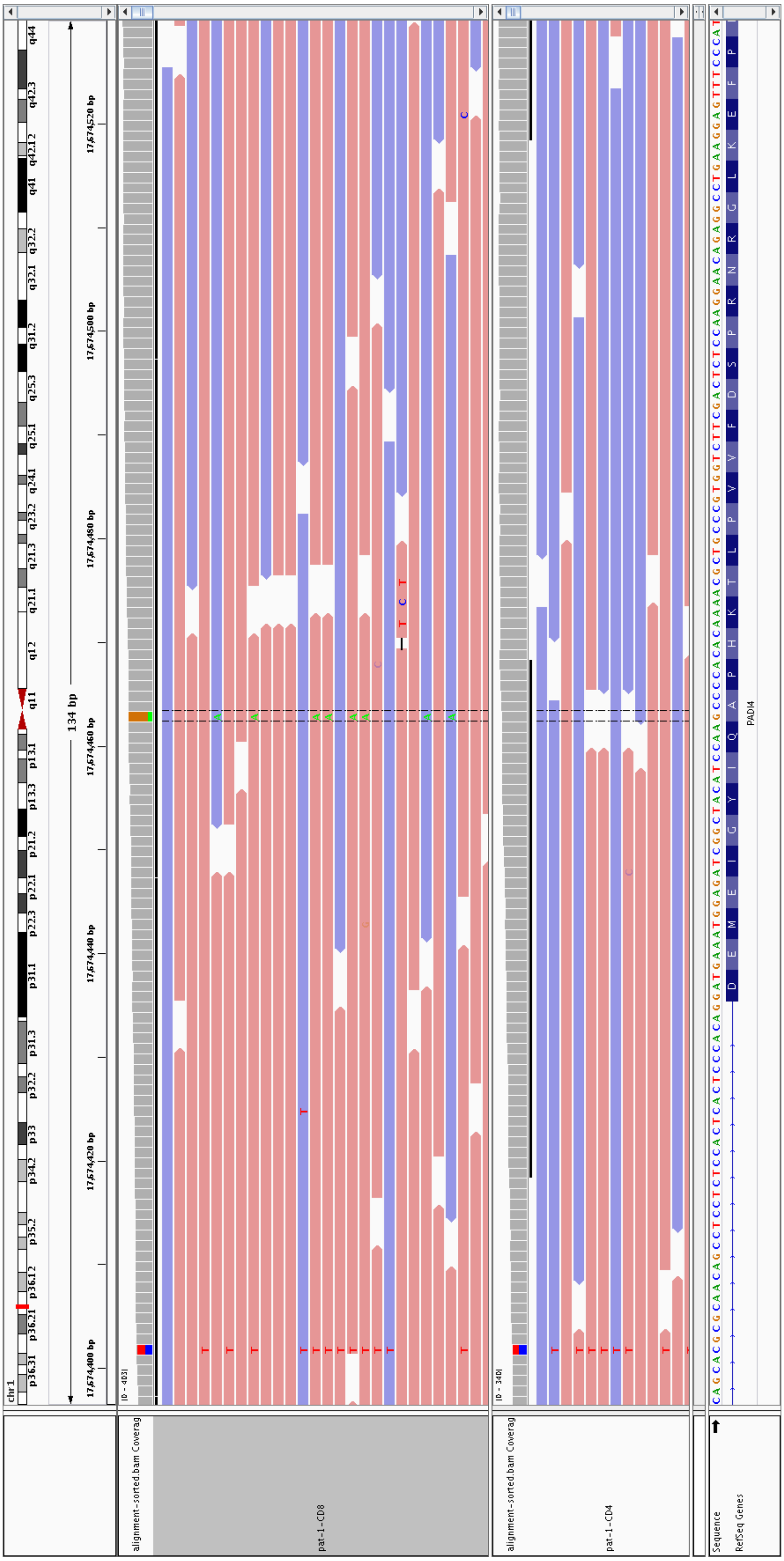
chr1
134 bp
alignment-sorted.bam Coverag
pat-1-CD8
pat-1-CD4
Sequence
RefSeq Genes
PADI4

B: SLAMF6

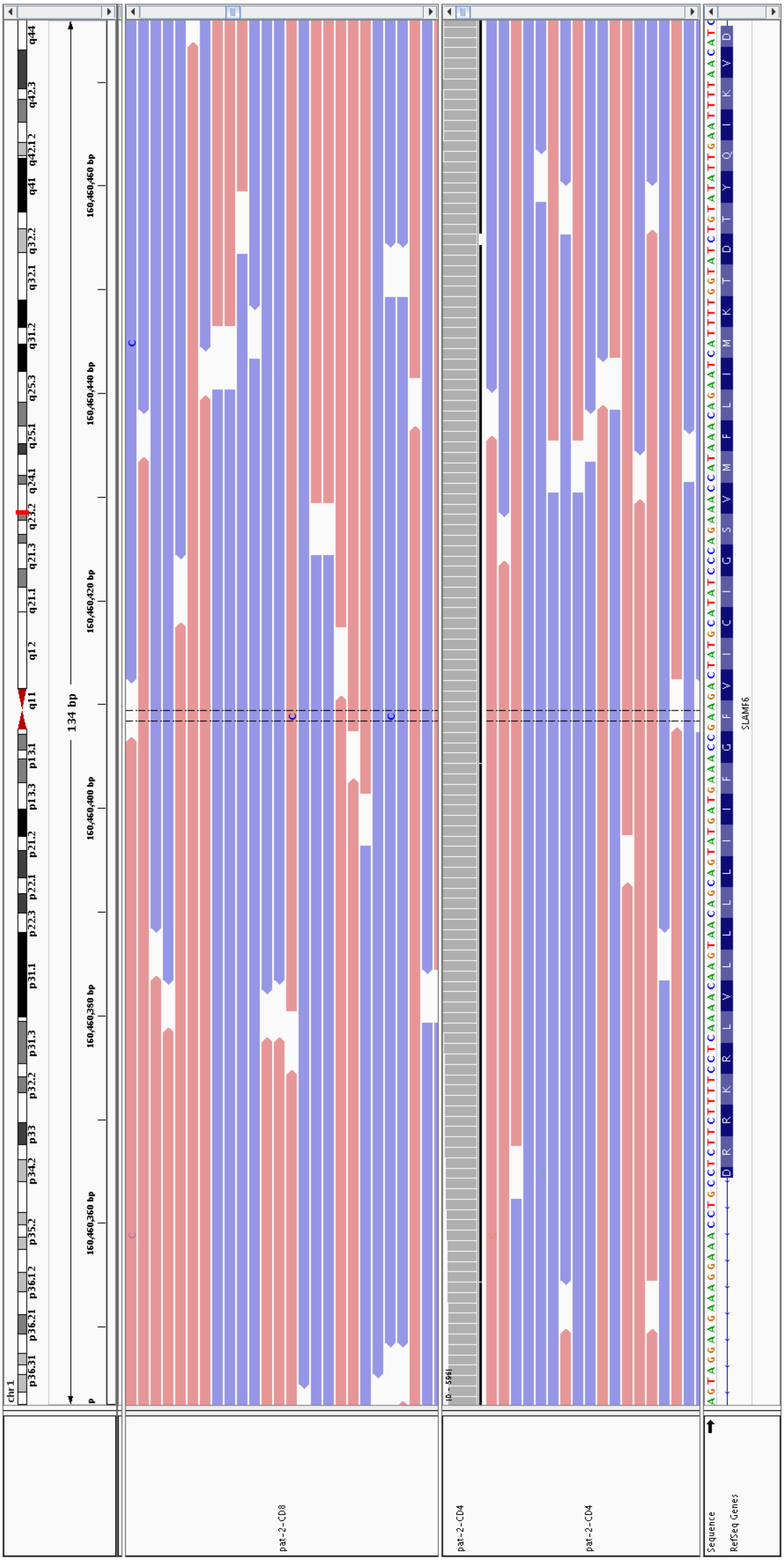

chr1
134 bp
160,460,360 bp
160,460,380 bp
160,460,400 bp
160,460,420 bp
160,460,440 bp
160,460,460 bp
pat-2-CD8
pat-2-CD4
pat-2-CD4
Sequence
RefSeq Genes
SLAMF6

C: IRF1

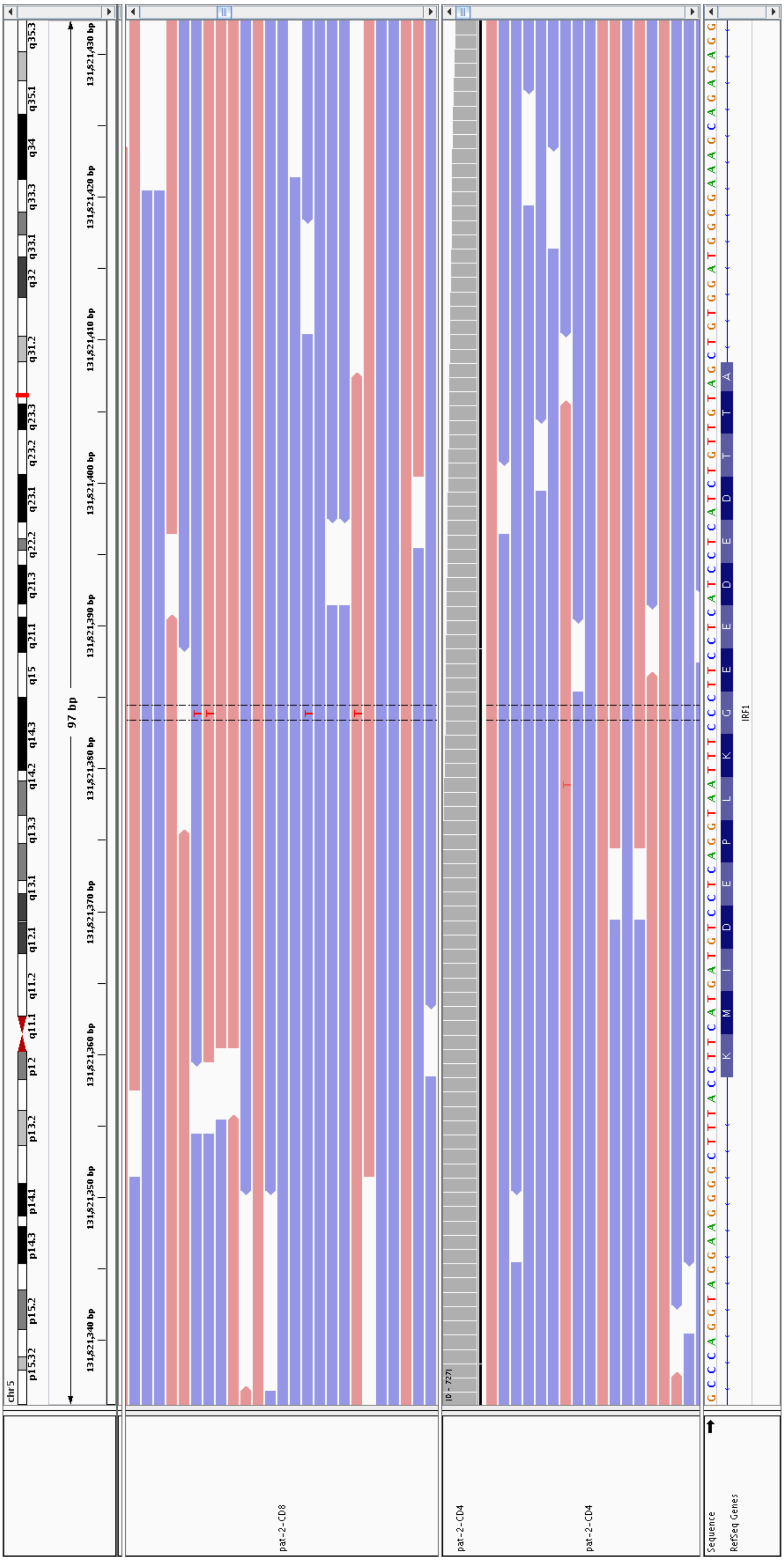

D: PTPRO

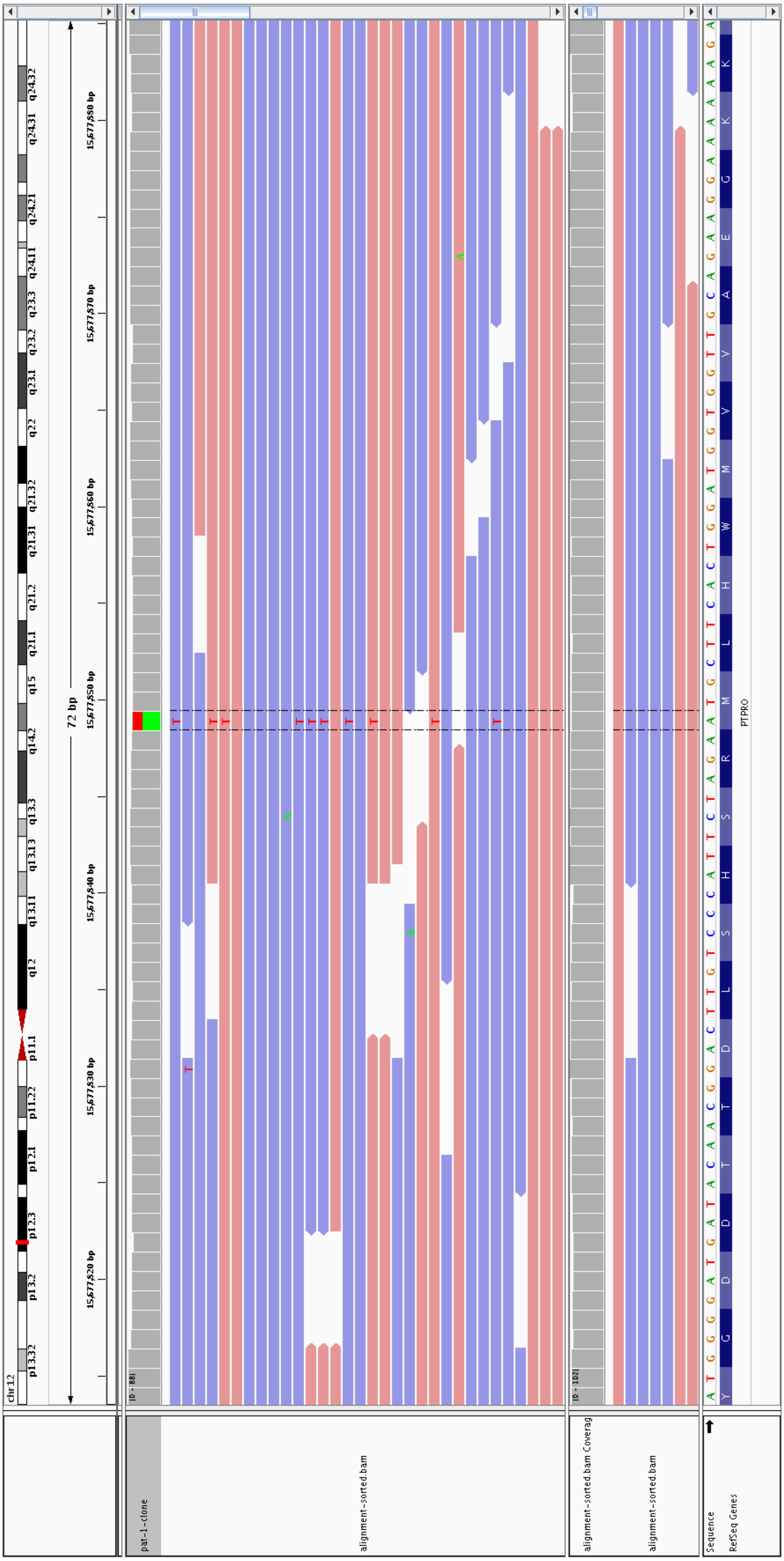
chr12
72 bp
15,677,820 bp
15,677,830 bp
15,677,840 bp
15,677,850 bp
15,677,860 bp
15,677,870 bp
15,677,880 bp
pat-1-clone
alignment-sorted.bam
alignment-sorted.bam Coverag
alignment-sorted.bam
Sequence
RefSeq Genes
PTPRO

E: CDK12

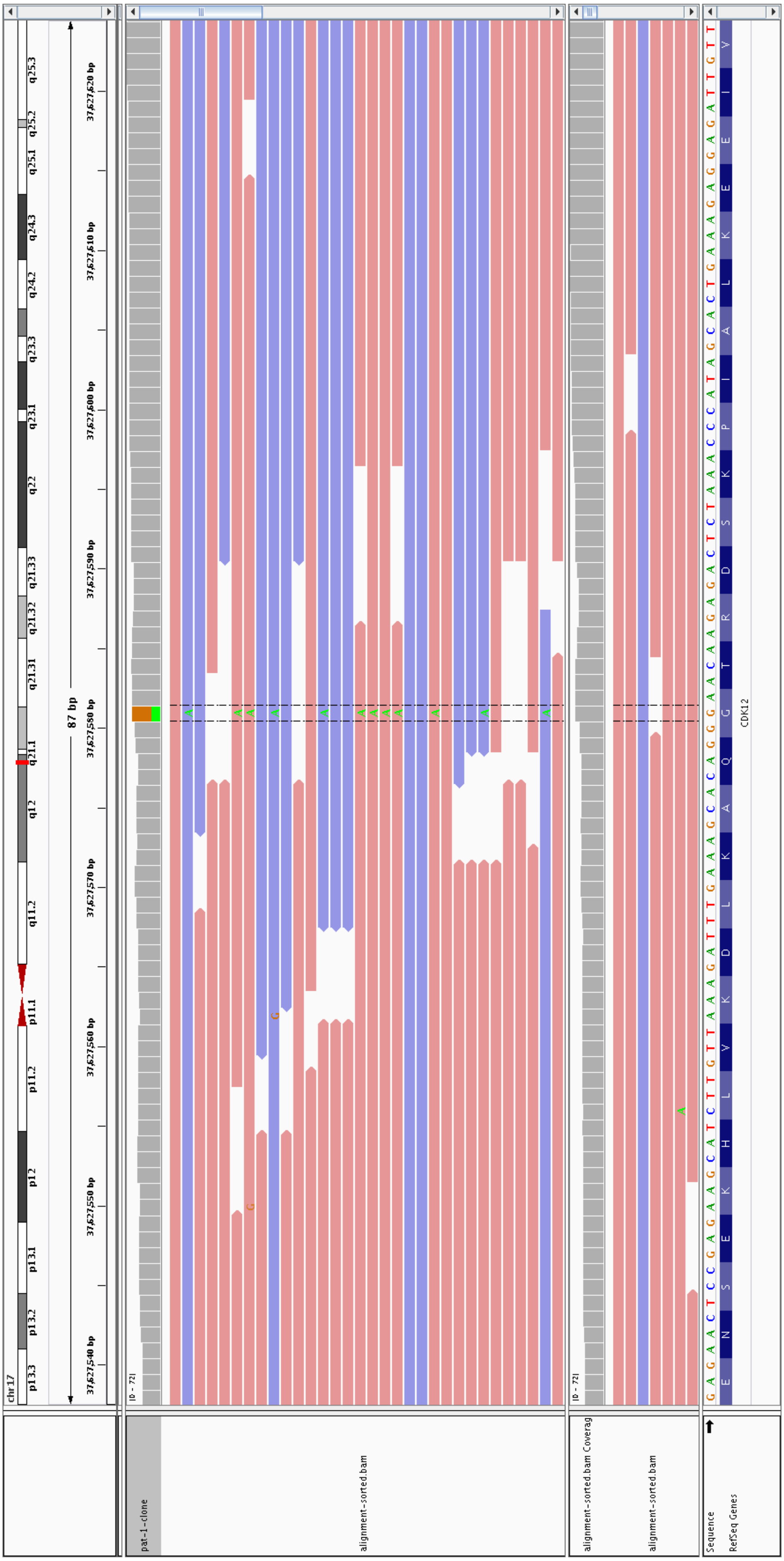
chr17
87 bp
pat-1-clone
alignment-sorted.bam
alignment-sorted.bam Coverag
alignment-sorted.bam
Sequence
RefSeq Genes
CDK12

F: PLRG1

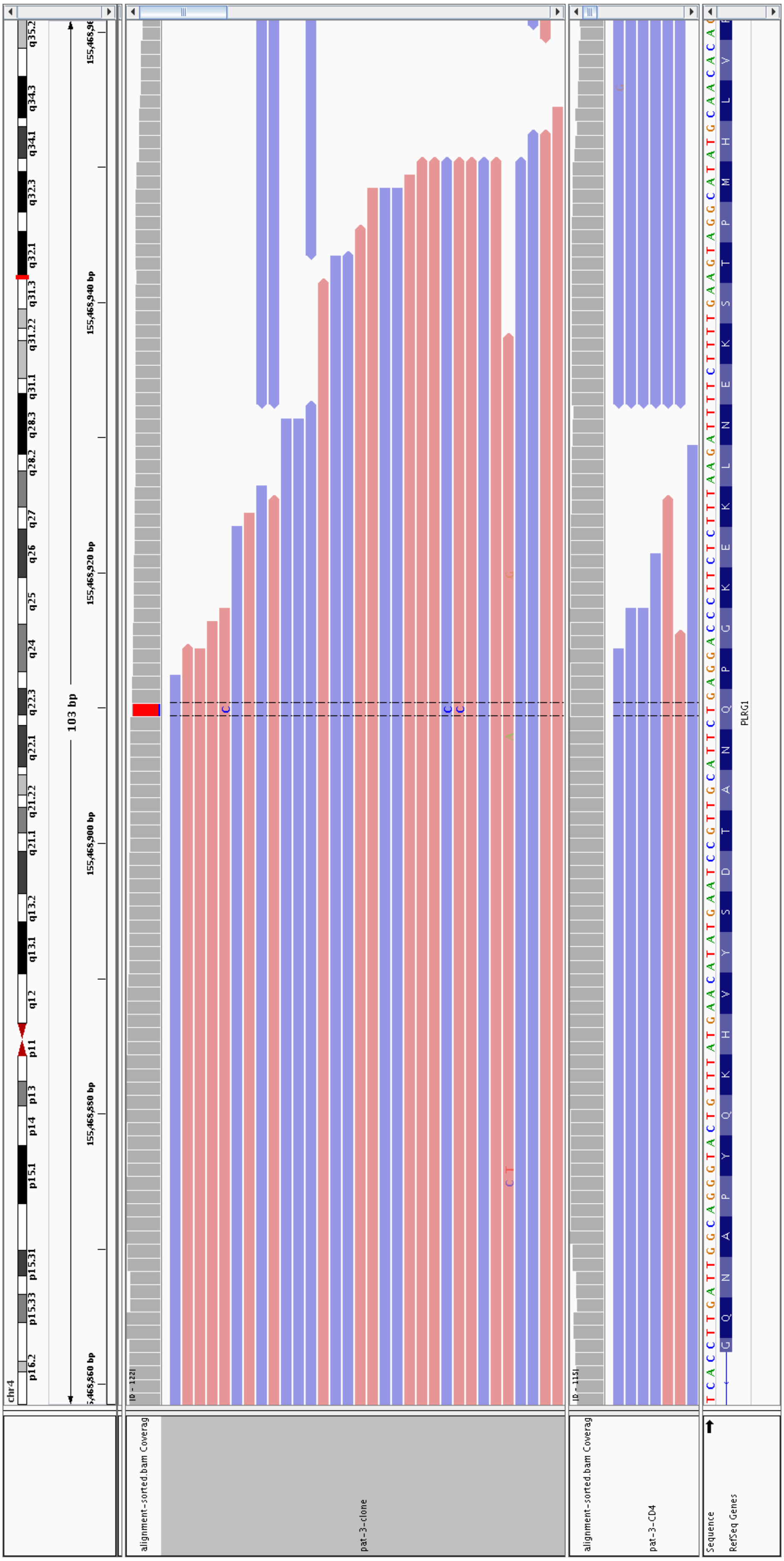
chr4
103 bp
alignment-sorted.bam Coverag
pat-3-clone
pat-3-CD4
Sequence
RefSeq Genes
PLRG1

Supplementary Figure 8. IGV visualization of example mutations. A-C: Point mutations in *PADI4* (Patient 1), *SLAMF6* (Patient 2) and *IRF1* (Patient 2) were identified using the immunopanel approach, In the left hand panel we display reads from the patient's CD8+ cells, while the right-side panel shows the reads from same patient's CD4+ cells. D-F: Exome sequencing identified somatic mutations in *PTPRO* (Patient 1), *CDK12* (Patient 1) and *PLRG1* (Patient 3). Here the left side displays the reads from the flow-cytometry sorted Vβ-stained CD8+ cells containing the mutated cells, and the CD4+ cells that served as germ-line control are shown on the right side. In the IGV screenshots only a representative sample of all aligned sequencing reads are presented (not all reads). More information on the mutations are presented in **Table 2** and **Supplementary table 6**.

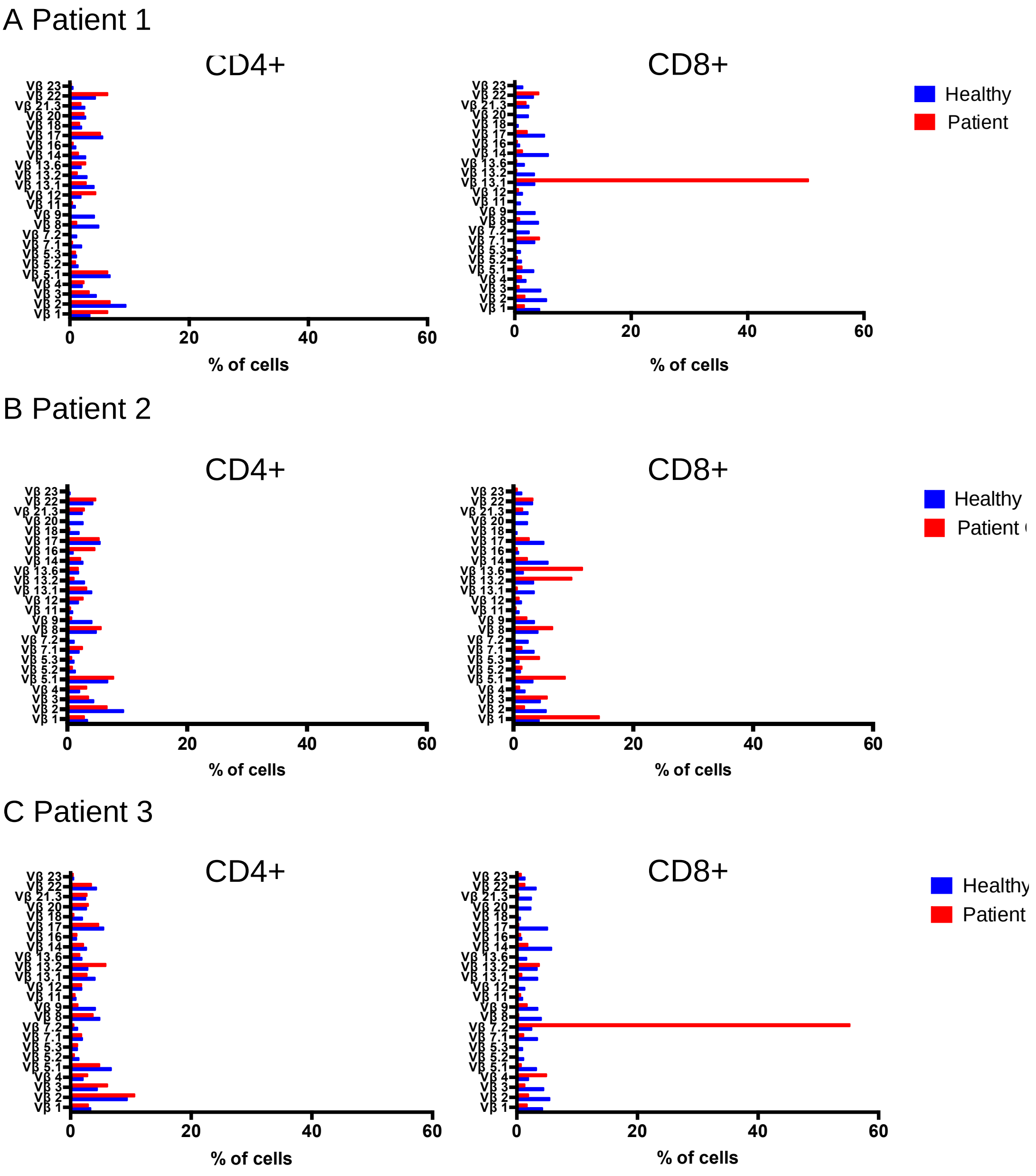

Supplementary Figure 9. Flow-cytometry analysis of expanded T-cell populations in patients 1, 2, and 3. RA patients' blood samples were screened for T-cell clonality with an antibody-based flow-cytometry assay. The antibodies target the Vβ region of the TCR, and thus recognize all T-cells using the same Vβ, even if they had a different TCR CD3R sequence. However, a skewed distribution in Vβ usage suggests that the sample has large T-cell clones. The figure shows the flow-cytometry Vβ screening results for patients 1 (A), 2 (B), and 3 (C).

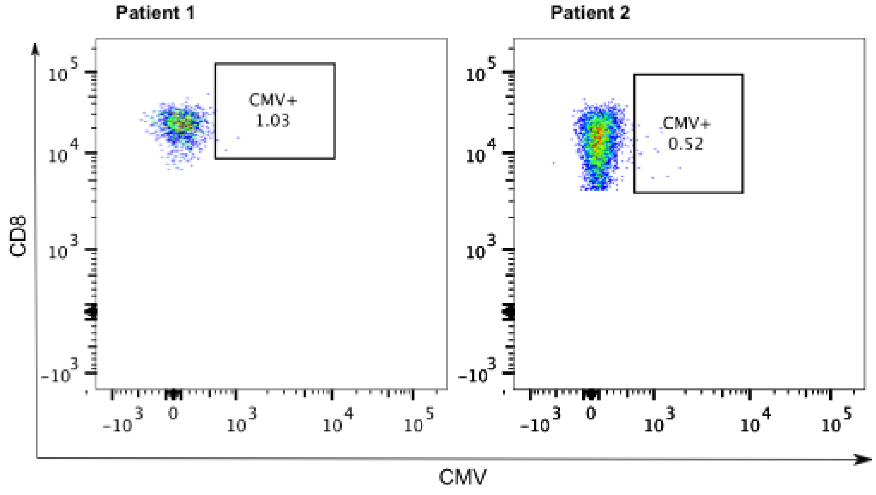

Supplementary Figure 10. The analysis of CMV-specific T cells with pentamer staining. To determine if the expanded CD8+ T-cell clones of patients 1 and 2 (who were HLA-A*0201-postive) were CMV-specific, cells were stained with CMV NLVPMVATV (HLA-A*0201) pentamer along with anti-CD3, anti-CD4, and anti-CD8 antibodies. Only 0.5-1% of CD8+ T cells were pentamer-positive, indicating that the expanded CD8+ T-cell clones of these two patients are not reactive to this CMV peptide.

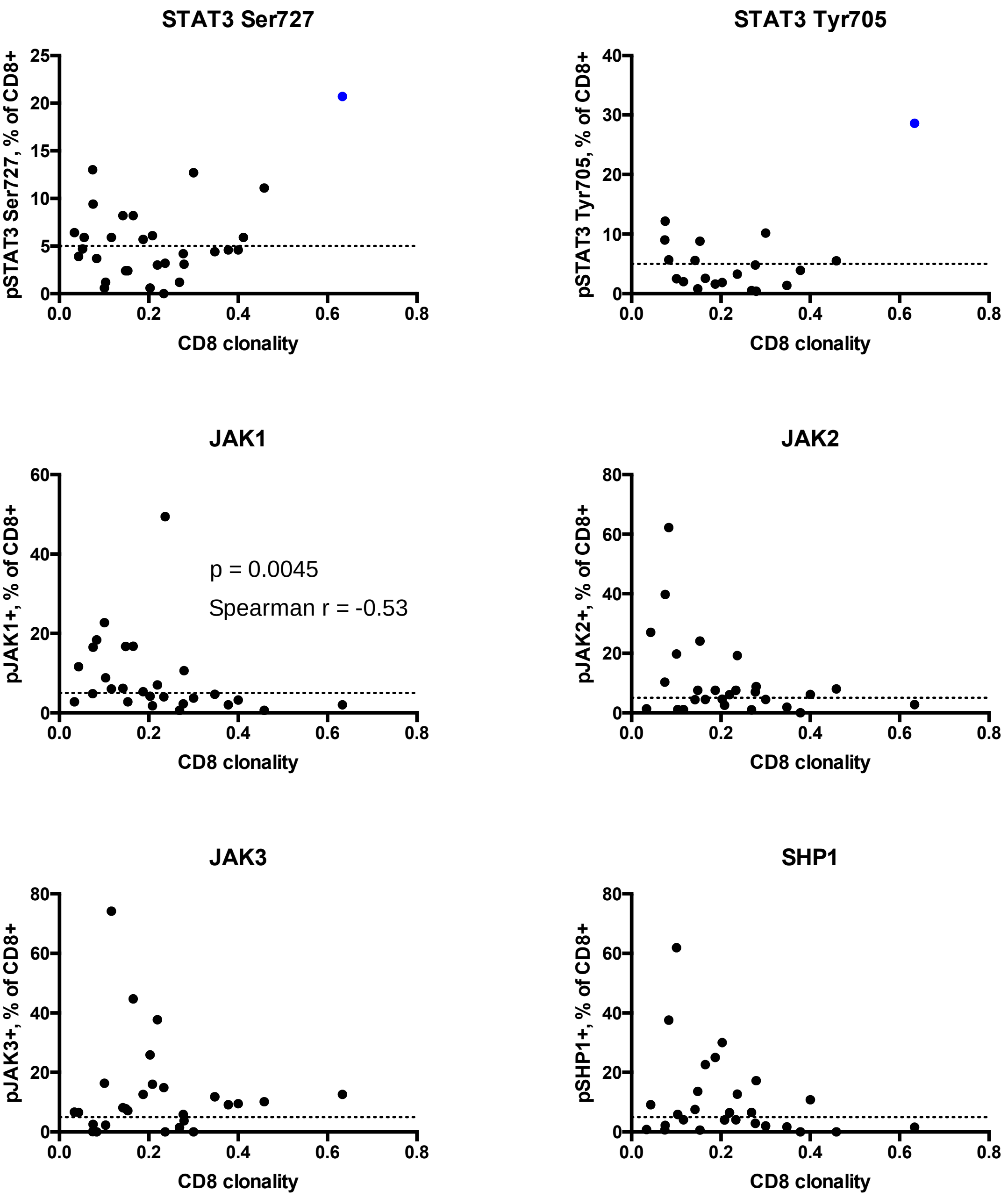

Supplementary Figure 11. Phosphorylation status of the JAK-STAT3 pathway in CD8+ T cells. Activation of the JAK-STAT pathway in RA patients was studied with phosphorylation-specific antibodies with flow cytometry. Healthy controls were used to determine the normal level of phosphorylation (<5% of cells phospho-positive, shown in plots with a dotted line). The correlation between clonality and the percent of phosphorylated CD8+ cells was tested with Spearman correlation (Spearman correlation coefficient r marked on plot if $p<0.05$). Statistically significant results are marked on the plot. Patient 1 showed remarkable STAT3 Ser727 and Tyr705 phosphorylation in CD8+ T cells, and is marked with blue in these plots.

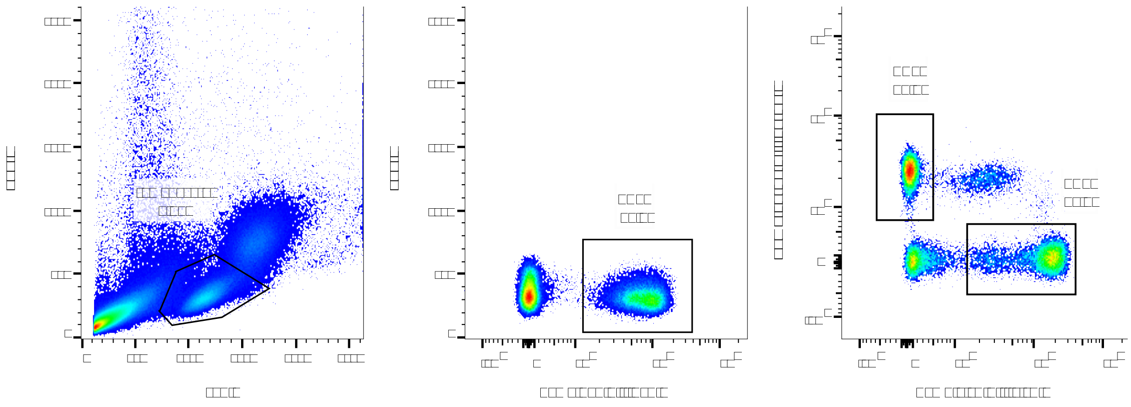

Supplementary Figure 12. Gating strategy for all PBMC cytometry experiments. As shown in upper left panel, the lymphocytes were first gated from the total PBMCs based on their morphological characters using SSC-A FSC-A plots. CD3+ cells were selected based on antibody staining (middle panel) and finally the CD3+ lymphocytes were separated into CD4+ and CD8+ lymphocytes as shown in the right hand panel. This gating strategy was applied in, sorting experiments as well as in the experiments where the lymphocytes' capacity to recognize the immunodominant CMV peptide (NLV).

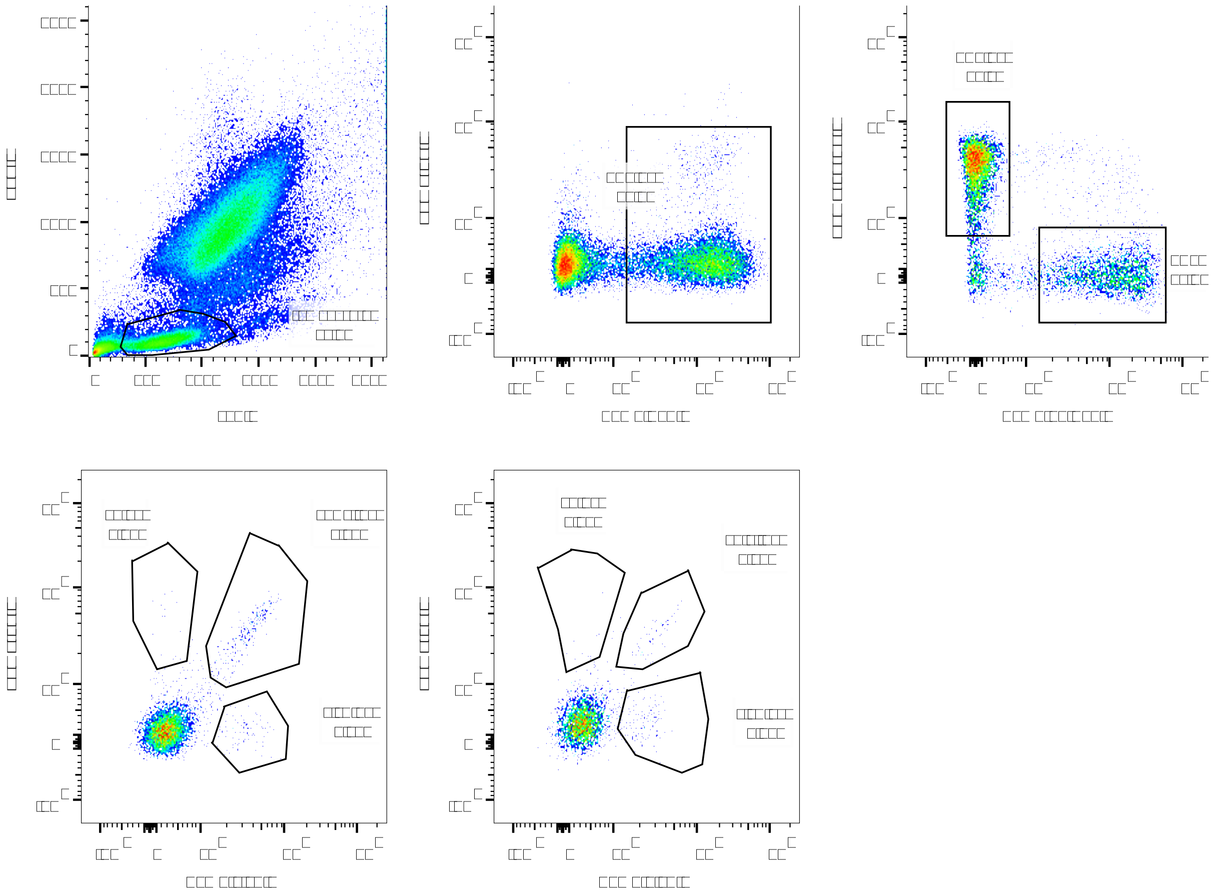

Vβ	Fluorochrome	Clone
Vb5.3	PE	3D11
Vb7.1	PE+FITC	ZOE
Vb3	FITC	CH92
Vb9	PE	FIN9
Vb17	PE+FITC	E17,5F3
Vb16	FITC	TAMAYA
Vb18	PE	BA62.6
Vb5.1	PE+FITC	IMMU157
Vb20	FITC	ELL1.4
Vb13.1	PE	IMMU222
Vb13.6	PE+FITC	JU74.3
Vb8	FITC	56C5.2
Vb5.2	PE	36213
Vb2	PE+FITC	MPB2D5
Vb12	FITC	VER2.32
Vb23	PE	AF23
Vb1	PE+FITC	BL37.2
Vb21.3	FITC	IGI25
Vb11	PE	C21
Vb22	PE+FITC	IMMU546
Vb14	FITC	CAS1.1.3
Vb13.2	PE	H132
Vb4	PE+FITC	WJF24
Vb7.2	FITC	ZIZOU4

Supplementary Figure 13. Gating strategy in screening experiments using the panel of T-cell receptor β variable chain (TCR Vβ) antibodies included in the IOTest Beta Mark TCR V kit. After red blood cell lysis, the lymphocytes from whole blood were first gated based on their morphological characters using

the SSC-A and FSC-A plots. CD3 positive lymphocytes (upper middle panel) were further divided into CD4+ and CD8+ populations using monoclonal antibodies listed in the materials and methods. Each sample was stained with all 8 antibody cocktails included in the IO test beta mark –staining kit and the β variable chain family was determined based on FITC and PE positivity from the CD8+ (lower left) and from the CD4+ (lower right) populations by comparing the antibody cocktail tube identifier to the list of β variable chain families indicated in the table provided by the manufacturer.

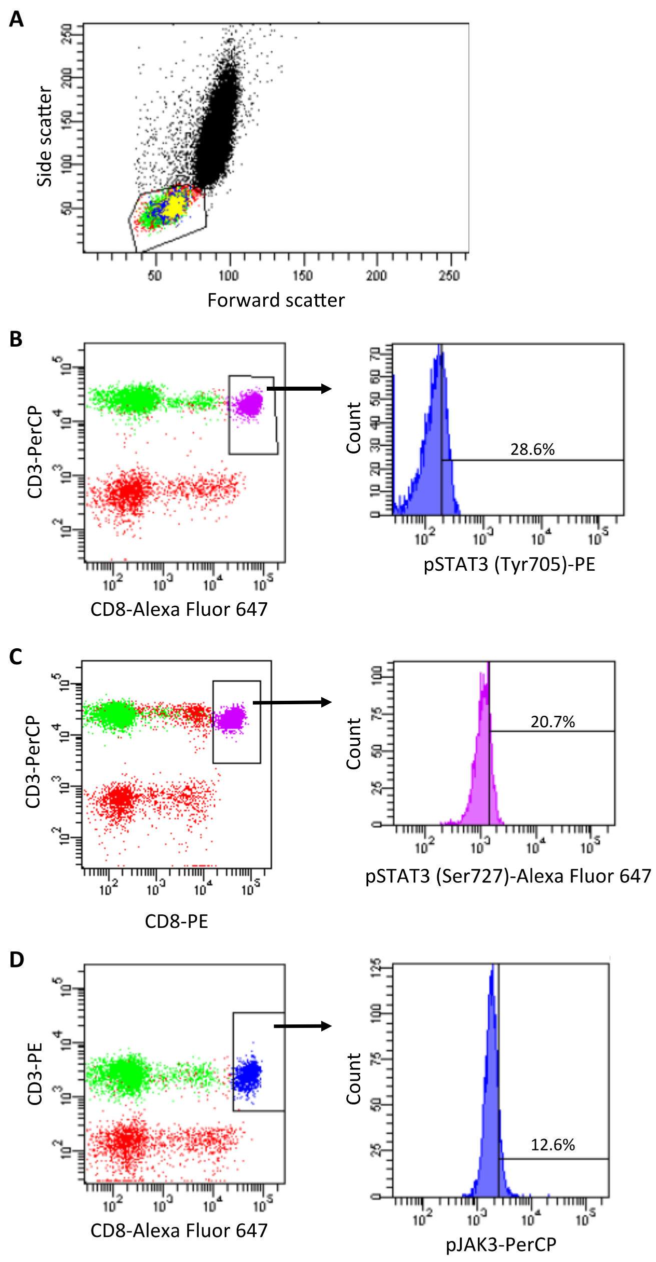

Supplementary Figure 14. Gating strategy for CD8+ T-cell JAK/STAT phospho-flow analysis. A) For all analyses, lymphocytes were first gated based on light scattering characteristics. B) For PE-linked phospho-antibodies (pSTAT3 Tyr705 shown, also pJAK1, pJAK2, pSHP-1), CD8-Alexa Fluor 647 and CD3-PerCP double positive lymphocytes were gated. C) For Alexa Fluor 647-linked pSTAT3 (Ser727) antibody, CD8-PE and CD3-PerCP double positive lymphocytes were gated. D) For PerCP-linked pJAK3 antibody, CD8-Alexa Fluor 647 and CD3-PE double positive lymphocytes were gated. After gating for CD3+CD8+ lymphocytes, the appropriate fluorescence intensity histograms were drawn. The gates were set to comprise <5% of the corresponding cells in a healthy control sample, and copied to the patient sample: the corresponding percentage of positive cells in the patient sample is shown (B-D).

	Patient ID	Clone size (% of CD8+)	AA sequence	nucleotide sequence	TCRBV gene	TCRBJ gene	TCRBD gene
Shared AA sequence	20	2.2	CASSPQRNTEAFF	CACCTACACACCCTGCAGCCAGAAGACTCGGCCCTGTATCTCTGCGCCAGCAGCCCCCAAAGGAACACTGAAGCTTTCTTTGGACAA	04-03*01	01-01*01	01
	79	2.2	CASSPQRNTEAFF	CACCTACACACCCTGCAGCCAGAAGACTCGGCCCTGTATCTCTGCGCCAGCAGCCCTCAGAGGAACACTGAAGCTTTCTTTGGACAA	04-03*01	01-01*01	01
Shared AA sequence	53	2.1	CSATRTARNQPQHF	GTGACCAGTGCCCATCCTGAAGACAGCAGCTTCTACATCTGCAGTGCAACGAGGACGGCTCGCAATCAGCCCCAGCATTTTGGTGAT	20-01	01-05*01	01
	79	9.1	CSATRTARNQPQHF	GTGACCAGTGCCCATCCTGAAGACAGCAGCTTCTACATCTGCAGTGCTACCAGGACGGCACGCAATCAGCCCCAGCATTTTGGTGAT	20-01	01-05*01	01
Shared AA sequence	17	8.0	CASSYPETQYF	CTGCTGGGGTTGGAGTCGGCTGCTCCCTCCCAAACATCTGTGTACTTCTGTGCCAGCAGTTATCCCGAGACCCAGTACTTCGGGCCA	06-02*01 or 06-03*01	02-05*01	unknown
	17	5.2	CASSYPETQYF	CTGCTGGGGTTGGAGTCGGCTGCTCCCTCCCAAACATCTGTGTACTTCTGTGCCAGCAGTTACCCGGAGACCCAGTACTTCGGGCCA	06-02*01 or 06-03*01	02-05*01	02-01
Shared AA sequence	7	4.9	CASSPSVSYEQYF	AACCTGAGCTCTCTGGAGCTGGGGGACTCAGCTTTGTATTTCTGTGCCAGCAGCCCCAGTGTTTCCTACGAGCAGTACTTCGGGCCG	09-01	01-01*01	02-07*01
	7	2.3	CASSPSVSYEQYF	AACCTGAGCTCTCTGGAGCTGGGGGACTCAGCTTTGTATTTCTGTGCCAGCAGCCCATCCGTGAGCTACGAGCAGTACTTCGGGCCG	09-01	02-01*02	02-07*01

Supplementary Table 1. Shared TCRB sequences in the study patients' CD8+ T-cell clones exceeding 1% of all CD8+. The amino-acid TCRB sequences of RA patients' CD8+ T-cell clones comprising over 1% of all CD8+ were compared. Two cases of TCRB sharing in these clones was observed (patients 20 and 79, and patients 53 and 79). Interestingly, two patients (17 and 7) harbored two >1% clones with the same amino-acid TCRB sequence in their CD8+ cells.

Patient ID	Gender	Largest CD8+ clone (% of CD8+)	Age at diagnosis	Hb	ESR	Leucocytes	Lymphocytes	Neutrophils	Monocytes	Eosinophils	Basophils	Thrombocytes	CRP	RF	Seropositive	ACPA	DAS28	tender joints	swollen joints	pat_global	rtg_erosions	HAQ	Duration
1	M	33.9	74.7	132	33	6.4	1.1	4.4	0.8	0.1	0.0	233	18	189	yes	160	3.46	5	0	3	no	2	5
2	F	7.8	72.0	126	47	7.1	2.1	4.1	0.6	0.3	0.1	460	2	19	yes	300	3.54	1	3	40	NA	1.3	NA
3	M	51.0	44.3	156	12	8.3	3.2	4.2	0.7	0.2	0.1	248	3	NA	yes	200	NA	11	1	55	yes	0.625	NA
4	F	13.2	73.6	150	31	7	2.2	4.1	0.6	0.1	0.0	337	6	10	no	6	NA	1	15	18	NA	0.63	NA
5	F	29.0	58.8	134	5	9.1	3.2	4.6	1.0	0.4	0.0	311	11	5	yes	204	NA	4	1	47	NA	NA	12
6	M	11.1	74.5	142	35	10.1	1.8	7.1	0.6	0.5	0.1	268	15	1.098	yes	300	4.03	3	4	50	no	0.8	12
7	M	13.8	66.1	141	17	6.7	1.8	3.6	0.5	0.8	0.0	195	7	128	yes	300	3.87	3	3	25	no	0.5	3
8	F	2.0	60.5	121	26	7.1	1.3	4.7	0.6	0.4	0.1	254	12	68	yes	60	3.09	2	5	23	yes	0.63	12
9	M	17.2	45.3	141	8	8.2	2.1	5.0	0.8	0.2	0.1	234	47	6	yes	130	4.03	7	3	80	yes	1.4	5
10	M	16.2	69.2	143	15	5.4	1.8	2.6	0.7	0.2	0.0	222	3	45	yes	114	3.32	1	1	42	yes	0.88	3
11	F	27.5	52.2	140	2	6.2	2.2	3.1	0.7	0.1	0.1	271	3	33	yes	50	2.13	9	5	9	no	0	6
12	M	15.7	50.6	135	8	5.6	1.5	3.2	0.4	0.4	0.1	226	3	100	yes	340	3.56	7	4	74	no	1.1	6
13	F	9.2	65.7	142	2	8.2	2.3	4.7	0.7	0.4	0.1	299	3	66	yes	301	2.79	11	4	47	yes	0	3
14	F	8.0	73.5	137	5	5.2	1.7	2.6	0.7	0.2	0.1	331	3	10	yes	46	4.54	22	10	47	no	0	2
15	F	4.5	25.0	115	8	4.5	1.1	2.7	0.6	0.0	0.0	204	4	4	no	6	4.8	15	7	84	no	1.75	2
16	F	6.7	62.4	143	27	6.1	2.4	3.2	0.4	0.1	0.0	302	4	252	yes	341	4.21	2	5	34	yes	0.5	12
17	F	20.3	70.7	111	57	5.5	1.2	3.7	0.5	0.1	0.0	406	63	14	no	18	6.02	16	24	20	NA	1.5	NA
18	F	10.2	65.1	131	30	8.2	2.4	5.0	0.6	0.3	0.0	239	7	122	yes	301	4.46	1	2	80	no	1	12
19	F	8.0	57.9	106	47	9.6	1.0	7.8	0.6	0.2	0.1	520	36	284	yes	255	5.21	6	17	65	NA	1.5	12
20	F	16.2	46.1	133	2	6.4	2.2	2.8	0.6	0.5	0.1	246	3	73	yes	301	2.1	7	8	18	no	0.13	5
21	F	3.0	48.2	124	16	9	1.8	6.7	0.4	0.2	0.0	279	9	0	no	0	5.03	13	12	7	no	0.375	3
22	F	7.0	68.2	130	8	7.3	2.6	4.0	0.5	0.2	0.1	319	2	263	yes	301	1.89	0	2	3	no	0	3
23	F	5.8	24.4	129	24	6.3	1.8	3.8	0.4	0.1	0.0	246	21	0	no	6	4.24	3	24	24	NA	0.125	NA
24	F	5.3	56.9	135	32	6.9	3.4	3.0	0.3	0.3	0.0	229	2	15	no	0	4.71	12	4	55	NA	0	NA
25	M	4.2	18.8	147	12	8.1	1.3	5.9	0.8	0.1	0.0	317	2	52	yes	340	3.83	9	6	NA	NA	NA	NA
26	F	1.2	47.5	144	17	7.4	1.5	5.1	0.6	0.1	0.0	340	8	18	yes	340	4.57	13	12	60	no	0	8
27	M	NA	64.7	137	7	5.3	NA	NA	NA	NA	NA	219	3	5	no	8	2.69	3	2	10	NA	0.38	6
28	F	1.5	30.6	131	35	9.8	3.0	5.9	0.6	0.2	0.0	248	15	25	yes	300	4.73	4	3	100	NA	2.25	2
29	F	19.4	32.9	130	5	6.2	2.7	2.7	0.6	0.2	0.1	275	4	11	no	7	3.18	15	7	30	NA	0.5	9
30	F	8.5	65.4	129	15	7.1	1.8	4.6	0.6	0.1	0.0	270	3	172	yes	38	3.31	5	5	9	NA	0	2
31	F	1.2	44.3	110	52	6.6	1.9	3.9	0.4	0.3	0.1	382	30	16	yes	300	3.73	0	10	20	no	1	2
32	F	5.2	39.3	123	24	5.7	1.4	3.6	0.4	0.3	0.1	329	6	29	yes	300	3.49	6	7	22	NA	0.38	4
33	F	6.1	53.4	117	63	6	2.0	3.2	0.5	0.2	0.0	179	3	65	yes	7	5.35	12	14	66	no	1.63	3
34	F	NA	56.1	140	2	6.5	3.1	3.0	0.3	0.1	0.0	211	3	64	yes	9	1.61	1	2	80	NA	0	12
35	F	NA	66.2	138	18	4.9	1.9	2.4	0.5	0.0	0.0	298	3	73	yes	7	3.21	1	3	10	no	0	2
36	F	NA	74.6	112	34	9.2	1.7	6.7	0.5	0.4	0.1	341	7	58	yes	51	3.57	0	7	26	no	0.38	6
37	F	2.6	47.0	123	86	8	1.6	5.7	0.6	0.1	0.1	485	9	53	yes	150	5.06	1	8	50	no	1	12
38	M	NA	53.3	144	26	16.1	5.5	9.0	1.1	0.3	0.0	410	12	143	yes	95	5.55	10	11	50	NA	1.88	2
39	F	NA	30.0	132	21	6.5	1.9	4.1	0.5	0.1	0.1	280	6	16	yes	220	3.04	2	2	5	no	0	12
40	F	11.2	64.0	140	5	5.7	1.9	3.2	0.3	0.2	0.1	237	3	188	yes	86	1.13	0	0	0	no	0	2

Patient ID	Gender	Largest CD8+ clone (% of CD8+)	Age at diagnosis	Hb	ESR	Leucocytes	Lymphocytes	Neutrophils	Monocytes	Eosinophils	Basophils	Thrombocytes	CRP	RF	Seropositive	ACPA	DAS28	tender joints	swollen joints	VAS	erosions	HAQ	Duration
41	F	1.0	38.4	128	5	4.8	1.4	2.5	0.4	0.3	0.0	235	3	25	no	26	2.11	2	5	30	NA	0.5	6
42	F	NA	39.5	120	5	8	1.5	5.3	1.1	0.1	0.1	223	3	16	no	7	4.74	24	15	65	no	1.13	4
43	M	NA	71.0	133	68	8.7	1.8	6.1	0.5	0.3	0.1	355	82	691	yes	300	7.12	25	25	50	yes	0.13	2
44	M	NA	73.1	140	19	8.9	2.1	5.7	0.8	0.2	0.0	277	3	20	no	7	2.76	0	1	30	no	0.63	NA
45	F	NA	65.6	134	15	6	1.8	3.0	0.7	0.5	0.1	303	4	40	yes	150	4.88	15	19	30	no	0.68	4
46	F	3.2	45.2	132	21	9.7	2.7	5.9	0.9	0.1	0.1	391	12	36	yes	340	4.79	17	1	NA	NA	NA	2
47	F	5.5	67.6	140	2	7.1	2.6	3.7	0.6	0.3	0.1	257	3	17	yes	180	1.65	1	7	15	NA	0	4
48	F	3.2	46.1	138	29	7.1	1.2	5.1	0.6	0.1	0.1	314	11	45	yes	300	4.01	1	10	33	no	1	2
49	M	1.8	29.9	144	5	6.4	2.2	2.7	0.5	0.9	0.1	292	3	354	yes	300	2.53	4	4	20	no	0	12
50	F	5.1	32.0	131	8	4.8	1.9	2.4	0.3	0.1	0.0	282	3	64	yes	180	3.72	11	2	82	no	0.25	3
51	F	8.1	49.9	140	5	7.5	1.7	5.2	0.5	0.2	0.0	231	4	18	yes	301	3.63	9	5	33	no	0.63	5
52	F	1.9	33.3	126	8	5.9	1.8	3.2	0.6	0.2	0.1	222	6	159	yes	340	1.67	3	2	15	NA	0	9
53	F	10.7	32.6	129	12	4.1	1.1	2.3	0.6	0.1	0.0	252	3	11	yes	300	4.46	12	9	65	no	0.86	4
54	F	5.1	35.3	129	58	7.6	1.3	5.4	0.5	0.4	0.1	414	43	0	no	0	NA	18	16	NA	NA	NA	NA
55	F	1.8	31.1	123	5	8	2.3	4.9	0.6	0.2	0.0	236	3	35	yes	341	5.05	27	13	75	no	2.25	NA
56	F	0.8	69.6	118	32	4.7	1.7	2.3	0.6	0.1	0.0	237	8	32	yes	6	5.57	13	10	70	NA	1	2
57	F	2.7	70.4	130	21	10.3	2.3	7.3	0.5	0.2	0.0	218	4	9	no	6	4.34	11	8	48	no	0.8	NA
58	F	5.3	49.3	127	5	6.5	1.9	3.3	0.5	0.7	0.1	306	3	121	yes	340	3.76	8	7	22	no	0.5	6
59	F	2.1	54.5	126	27	11.6	2.1	7.9	1.0	0.5	0.0	467	10	199	yes	340	5.08	6	23	49	no	0.5	6
60	F	6.8	62.6	134	48	10.1	2.0	6.4	1.1	0.5	0.0	328	45	251	yes	301	4.94	6	7	37	NA	0.5	1
61	F	NA	33.5	112	24	7.5	2.0	4.1	0.8	0.6	0.0	279	2	59	yes	341	4.53	12	10	45	no	0.5	4
62	F	13.6	36.6	131	12	7.5	2.8	3.3	0.6	0.8	0.1	287	2	26	yes	150	3.08	6	7	21	no	0	6
63	F	7.7	63.0	128	38	10	2.9	4.6	1.6	0.8	0.1	500	25	73	yes	301	3.17	2	7	10	no	0.25	12
64	F	NA	65.8	117	54	6.8	1.3	4.8	0.3	0.4	0.0	297	8	212	yes	301	4.95	3	11	25	no	1	12
65	F	8.9	37.8	118	9	5.9	1.5	3.7	0.5	0.2	0.1	259	2	23	yes	49	2.54	6	3	15	NA	0.25	NA
66	F	25.0	65.5	130	13	5.1	1.3	3.1	0.5	0.3	0.0	245	2	41	yes	44	3.82	1	8	70	no	0.375	5
67	M	NA	68.0	114	59	12.1	3.1	7.4	1.1	0.4	0.1	405	65	8	no	6	NA	20	16	NA	yes	NA	2
68	F	4.6	64.3	117	40	8.8	1.9	5.9	0.7	0.2	0.1	374	95	8	no	6	7.09	20	28	70	no	2.1	2
69	F	20.8	81.6	124	16	5.6	1.4	3.5	0.5	0.2	0.1	175	2	79	yes	301	4.46	4	4	60	no	2.5	2
70	M	3.5	47.0	150	21	7.7	3.3	3.1	1.0	0.2	0.1	225	13	99	yes	301	4.99	8	17	40	no	0.25	12
71	F	NA	52.2	150	17	12.8	4.4	6.9	0.9	0.0	0.0	233	17	25	yes	150	4.02	2	2	77	NA	0.75	3
72	F	1.7	61.6	133	76	11.6	2.6	7.5	0.9	0.5	0.1	444	27	289	yes	26	6.68	15	7	72	NA	1.88	NA
73	F	NA	63.0	115	30	9.2	1.5	6.6	0.7	0.3	0.0	188	20	15	no	19	4.71	16	5	42	yes	1	7
74	F	3.5	43.4	149	5	8.1	1.5	5.9	0.6	0.0	0.0	216	2	256	yes	301	2.87	8	8	15	no	0.625	7
75	M	4.7	54.8	141	20	5.9	2.5	2.2	0.7	0.4	0.1	222	2	489	yes	301	NA	6	14	NA	no	NA	2
76	F	0.9	27.5	124	13	7.9	2.0	4.8	0.8	0.3	0.1	269	2	79	yes	301	4.09		10	26	yes	0	10
77	F	NA	68.3	124	7	10.9	3.5	6.3	0.5	0.3	0.1	260	9	35	yes	301	2.13	0	4	55	NA	0.5	12
78	F	NA	39.1	142	8	9.2	2.1	6.1	0.8	0.8	0.0	232	3	120	yes	340	4.65	14	5	68	NA	1.38	NA
79	F	10.3	76.6	113	68	8.1	1.7	5.5	0.7	0.1	0.0	319	25	11	no	6	5.17	1	5	50	no	0.88	8
80	F	2.0	44.2	146	27	7.8	2.1	5.0	0.6	0.1	0.1	349	11	18	yes	301	3.87	2	2	27	yes	1	14
81	F	19.1	59.0	122	24	5.1	1.5	2.9	0.5	0.1	0.2	273	5	216	yes	120	5.28	13	6	92	NA	0.5	NA

Patient ID	Gender	Largest CD8+ clone (% of CD8+)	Age at diagnosis	Hb	ESR	Leucocytes	Lymphocytes	Neutrophils	Monocytes	Eosinophils	Basophils	Thrombocytes	CRP	RF	Seropositive	ACPA	DAS28	tender joints	swollen joints	pat_global	rtg_erosions	HAQ	Duration
82	F	11.2	52.3	128	19	7.1	1.7	4.7	0.4	0.1	0.2	319	2	79	yes	180	5.12	19	18	39	NA	1	NA
Av		9.3	53.6	131	23.4	7.5	2.1	4.5	0.6	0.3	0.0	292	12.2	101		176.9	4.0	7.8	7.9	41.2		0.7	6.0
Med		6.7	54.7	131	18.5	7.1	1.9	4.4	0.6	0.22	0.06	274	4	45		180	4.0	6	7	40		0.6	5
Min		0.78	18.8	106	2	4.1	0.96	2.2	0.3	0	0	175	2	0		0	1.13	0	0	0		0	1
Max		51.0	81.6	156	86	16.1	5.474	9.0	1.6	0.9	0.2	520	95	1,098		341	7.12	27	28	100		2.5	14

Supplementary Table 2. Clinical characteristics of all study patients.
The study comprised of 82 patients (66 female, 16 male). Leukocyte, neutrophil, lymphocyte, monocyte, eosinophil, basophil, and thrombocyte counts are presented as 10E9/l. Abbreviations: CD8+ clone, the size (% of CD8+) the largest CD8+ T-cell clone; dg, diagnosis; Av, average of values in column; Med, median of values in column; Min, smallest value in column; Max, largest value in column; Hb, hemoglobin level (g/l); ESR, erythrocyte sediment rate (mm/h); CRP, C-reactive protein (mg/l), RF, rheumatoid factor (IU/ml); ACPA, anti-citrullinated peptide antibody (U/ml); DAS28, Disease Activity Score in 28 joints; VAS, patient's global assessment of disease activity on visual analogue scale; Erosions, joint erosions detected in hand or feet x-rays at diagnosis; HAQ, Health Assessment Questionnaire; Duration, duration of symptoms (months) before diagnosis; NA, not available. The total numbers of tender and swollen joints (not only counts of joints included in DAS28 score) are shown in separate columns.

Pat. ID	**HLA-A_1**	**HLA-A_2**	**HLA-B_1**	**HLA-B_2**	**HLA-C_1**	**HLA-C_2**	**HLA-DRB1_1**	**HLA-DRB1_2**
1	02:01	03:01	40:01	40:01	03:04	03:04	04:04	15:01
2	03:01	24:02	15:01	35:01	03:03	04:01	01:01	01:01
3	03:01	24:02	07:02	08:01	03:04	07:01	01:01	03:01
4	02:01	02:01	07:02	40:01	02:02	07:02	13:02	15:01
5	11:01	31:01	18:01	35:01	03:03	07:01	08:01	15:01
6	02:01	03:01	15:01	15:01	03:04	03:04	04:01	04:01
7	02:01	02:01	27:05	51:01	01:02	15:02	09:01	13:01
8	01:01	24:02	08:01	40:01	03:04	07:01	03:01	13:02
9	02:01	03:01	15:01	18:01	03:03	07:01	04:01	08:01
10	32:01	68:01	08:01	35:01	04:01	07:01	01:01	03:01
11	02:01	26:01	51:01	18:01	01:02	14:02	01:01	13:01
12	24:02	32:01	35:01	35:01	03:03	04:01	01:01	04:01
13	01:01	02:01	08:01	15:01	04:01	07:01	04:01	08:01
14	03:01	03:01	35:01	39:01	04:01	07:02	04:01	08:01
15	03:01	24:02	18:01	35:01	04:01	07:01	01:01	04:04
16	03:01	03:01	35:01	35:01	04:01	04:01	01:01	01:01
17	24:02	68:01	08:01	40:02	02:02	07:01	01:01	03:01
18	24:02	32:01	15:01	56:01	01:02	03:04	04:01	04:01
19	02:01	02:01	07:02	40:01	03:04	07:02	13:02	15:01
20	02:01	03:01	07:02	40:02	03:04	07:02	14:02	15:01
21	01:01	68:01	08:01	35:01	03:03	07:01	03:01	08:01
22	02:01	26:01	15:01	40:02	03:04	04:01	15:01	15:01
23	NA	NA	NA	NA	NA	NA	NA	NA
24	02:01	03:01	40:01	47:01	03:04	06:02	09:01	15:01
25	02:01	02:01	13:02	15:01	04:01	06:02	01:01	10:01
26	02:01	23:01	27:05	44:03	01:02	04:01	04:08	07:01
28	03:01	33:03	35:01	58:01	03:02	04:01	01:01	03:01
29	32:01	68:01	35:01	44:02	04:01	07:04	01:01	04:01
30	01:01	11:01	35:01	44:02	01:02	04:01	01:01	01:01
31	02:01	32:01	15:01	44:02	03:04	05:01	04:01	13:03
32	02:01	24:02	44:02	44:02	05:01	05:01	04:01	04:01
33	02:01	03:01	15:01	35:03	03:04	04:01	04:01	15:01
37	02:01	68:01	35:01	51:01	04:01	15:02	12:01	13:01
40	03:01	24:02	07:02	27:05	01:02	07:02	04:01	04:08
41	02:01	03:01	27:05	35:01	01:02	04:01	01:01	09:01
46	02:01	32:01	15:01	35:01	03:03	04:01	04:01	04:01
47	02:01	03:01	35:01	56:01	01:02	04:01	01:01	04:01
48	02:01	31:01	27:05	56:01	01:02	01:02	01:01	04:08
49	02:01	03:01	15:01	15:01	03:04	03:04	04:01	04:01
50	03:01	32:01	35:01	44:02	04:01	05:01	01:01	04:01
51	02:01	02:01	18:01	27:05	01:02	07:01	01:01	04:01
52	02:01	03:01	15:01	27:05	01:02	03:03	01:01	04:01
53	02:01	03:01	15:01	35:01	03:03	04:01	01:01	04:01
54	03:01	68:01	07:02	08:01	07:01	07:02	03:01	15:01
55	02:01	24:02	39:01	39:01	07:02	12:03	04:04	12:01
56	11:01	23:01	07:02	49:01	07:01	07:02	03:01	13:01
57	02:01	02:01	13:02	18:01	06:02	07:01	07:01	14:01
58	32:01	68:01	15:01	40:01	03:03	03:04	04:04	11:01
59	24:02	25:01	15:01	18:01	03:03	12:03	01:01	01:01
60	03:01	03:01	35:01	44:27	04:01	07:04	01:01	16:01
61	24:02	32:01	08:01	39:01	07:01	07:02	03:01	04:04
62	24:02	32:01	07:02	35:01	04:01	07:02	04:01	15:01
63	02:01	03:01	15:01	27:05	01:02	03:03	01:01	15:01
65	02:01	03:01	35:01	44:02	04:01	05:01	01:01	04:01
66	02:01	02:01	27:05	44:02	01:02	05:01	04:08	12:01
68	01:01	02:01	08:01	40:01	03:04	07:01	03:01	13:02
69	02:01	03:01	15:01	18:01	04:01	07:01	04:04	15:01
70	03:01	03:01	07:02	35:01	04:01	07:02	04:04	15:01
72	03:01	30:01	07:02	13:02	03:04	06:02	01:01	10:01
74	02:03	11:01	39:09	40:02	07:02	07:02	04:05	04:05
75	02:01	03:01	44:02	57:01	06:02	07:04	07:01	16:01
76	01:01	01:01	08:01	39:01	07:01	12:03	01:01	03:01
79	02:01	68:01	07:02	27:05	01:02	07:04	01:01	01:01
80	02:01	03:01	07:02	15:01	03:04	07:02	04:01	15:01
81	03:01	31:01	15:01	35:01	03:03	04:01	01:01	10:01
82	24:02	68:01	07:02	39:01	07:02	07:02	04:04	14:01

HC ID	**HLA-A_1**	**HLA-A_2**	**HLA-B_1**	**HLA-B_2**	**HLA-C_1**	**HLA-C_2**	**HLA-DRB1_1**	**HLA-DRB1_2**
HC1	02:01	03:01	35:03	40:01	03:04	04:01	04:03	08:01
HC2	02:01	02:01	15:01	15:18	03:04	07:04	04:01	04:04
HC3	01:01	24:02	08:01	39:01	07:01	07:02	08:01	12:01
HC4	01:01	03:01	07:02	08:01	07:01	07:02	03:01	15:01
HC5	02:01	24:02	07:02	27:05	01:02	07:02	04:04	15:01
HC6	01:01	02:01	44:02	57:01	05:01	06:02	07:01	15:01
HC7	02:01	31:01	40:01	51:01	01:02	03:04	04:04	08:01
HC8	02:01	33:03	35:01	58:01	03:02	04:01	01:01	13:02
HC9	24:02	24:02	40:01	51:01	03:04	14:02	09:01	11:01
HC10	03:01	03:01	07:02	18:01	07:01	07:02	01:01	04:04
HC11	02:01	02:01	13:02	15:01	04:01	06:02	07:01	08:01

HC12	02:01	03:01	27:05	35:01	02:02	04:01	01:01	08:01
HC13	02:01	68:01	08:01	15:01	04:01	07:01	04:04	08:01
HC14	02:01	02:01	27:05	44:02	02:02	05:01	04:04	12:01
HC15	02:01	02:01	15:01	27:05	02:02	03:03	08:01	13:01
HC16	02:01	11:01	18:01	55:01	03:03	07:01	04:04	14:54
HC17	02:01	26:01	27:05	40:02	01:02	03:04	01:01	08:02
HC18	02:01	02:01	13:02	15:01	03:03	06:02	07:01	13:01
HC19	01:01	03:01	35:01	37:01	04:01	06:02	01:01	12:01
HC20	03:01	03:01	07:02	35:01	04:01	07:02	01:01	15:01

Supplementary Table 3. HLA typing of RA patients. HLA-A, HLA-B, HLA-C, and HLA-DRB1 loci were typed from 65 newly diagnosed RA patients and 20 healthy controls. Patients sequenced with immunogene-panel sequencing are listed first in the table, followed by other RA patients and healthy controls. Abbreviations: HC, healthy control; NA, not available.

A2M	CCL25	CD79A	CXCL5	GZMA	IL19	KIR2DL1	MPL	PSIP1	SOD3	WIPF1
A2ML1	CCL26	CD79B	CXCL6	GZMB	IL1A	KIR2DL3	MPO	PSMB10	SPN	VPREB1
ABCB1	CCL27	CD80	CXCL9	GZMK	IL1B	KIR2DL4	MR1	PSMB5	SPP1	XCL1
ABCF1	CCL28	CD81	CXCR1	GZMM	IL1F10	KIR2DS4	MRC1	PSMB6	SRC	XCL2
ACE	CCL3	CD82	CXCR2	HAMP	IL1R1	KIR3DL1	MRC2	PSMB7	ST6GAL1	XCR1
ADA	CCL3L1	CD83	CXCR3	HCK	IL1R2	KIR3DL2	MRE11A	PSMB8	STAT1	XRCC5
ADAM10	CCL3L3	CD84	CXCR4	HGF	IL1RAP	KIR3DL3	MS4A1	PSMB9	STAT2	YES
ADAM17	CCL4	CD86	CXCR5	HLA-A	IL1RAPL1	KIT	MS4A3	PSME1	STAT3	YWHAZ
ADAM8	CCL4L1	CD8A	CXCR6	HLA-B	IL1RAPL2	KITLG	MS4A5	PSME2	STAT4	ZAP70
AGFG1	CCL4L2	CD8B	CXCR7	HLA-C	IL1RL1	KLRB1	MSR1	PSME3	STAT5A	ZEB1
AICDA	CCL5	CD9	CYBA	HLA-DMA	IL1RL2	KLRC1	MST1	PSMF1	STAT5B	ZFP36
AIMP1	CCL7	CD93	CYBB	HLA-DMB	IL1RN	KLRC2	MST1R	PSTPIP1	STAT6	ZFP36L1
AIRE	CCL8	CD96	CYSLTR1	HLA-DOA	IL2	KLRD1	MTOR	PTAFR	TAGAP	
ALCAM	CCND2	CD97	CYTL1	HLA-DOB	IL20	KLRK1	MUC1	PTEN	TAL1	
ALK	CCND3	CD99	DARC	HLA-DPA1	IL20RA	KRAS	MX1	PTGER4	TANK	
ANP32B	CCNE1	CD99L2	DCD	HLA-DPB1	IL21	L1CAM	MYC	PTGFRN	TAP1	
ANPEP	CCR1	CDH5	DCLRE1C	HLA-DQA1	IL21R	LAG3	MYD88	PTGS2	TAP2	
ANXA6	CCR10	CDK6	DDR1	HLA-DQB1	IL22	LAIR1	MYLK	PTK2	TAP2	
ATM	CCR2	CDKN1A	DEFA1	HLA-DQB2	IL22RA1	LAIR2	NCAM1	PTK2B	TAPBP	
B2M	CCR3	CEACAM1	DEFA3	HLA-DRA	IL22RA2	LAMP1	NCF1	PTPN11	TBK1	
BACH2	CCR4	CEACAM3	DEFA4	HLA-DRB5	IL23A	LAMP2	NCF2	PTPN2	TBX21	
BANK1	CCR5	CEACAM5	DEFA5	HLA-E	IL23R	LAMP3	NCF4	PTPN22	TCF3	
BATF	CCR6	CEACAM6	DEFA6	HLA-F	IL24	LAMTOR3	NCR1	PTPN6	TCF7	
BAX	CCR6	CEACAM8	DEFB1	HMGB1	IL25	LAX1	NCR2	PTPRC	TCN2	
BCAM	CCR7	CEBPB	DEFB103A	HMMR	IL26	LCK	NCR3	PTPRCAP	TDP2	
BCAP31	CCR9	CEBPE	DEFB105A	HPS3	IL27	LCP2	NDUFS3	PTPRE	TEK	
BCL2	CCRL1	CFD	DEFB106A	HRAS	IL27RA	LEAP2	NFATC1	PTPRJ	TFRC	
BCL2L1	CCRL2	CFH	DEFB119	HRH2	IL28A	LEP	NFATC2	PVR	TGFB1	
BCL6	CCRN4L	CFHR1	DEFB123	HRH4	IL28B	LIF	NFATC3	PVRL1	THBD	
BIRC3	CD101	CFHR2	DEFB4A	HSP90B1	IL28RA	LIFR	NFATC4	PVRL2	THY1	
BLK	CD109	CFHR3	DKC1	HSPA4	IL29	LIG1	NFIL3	PXK	TICAM1	
BLM	CD14	CFHR4	DOCK2	HSPA6	IL2RA	LIG4	NFKB1	RAC1	TICAM2	
BMP2	CD151	CFHR5	DPP4	HSPD1	IL2RB	LILRA1	NFKB2	RAC2	TIMP1	
BSG	CD160	CFI	DUSP1	HTN3	IL2RG	LILRA2	NFKBIA	RAG1	TIRAP	
BST1	CD163	CFLAR	EBF1	ICAM1	IL3	LILRA3	NFKBIB	RAG2	TLR1	
BST2	CD164	CFP	EBF2	ICAM2	IL31	LILRA4	NFKBIE	RBPJ	TLR10	
BTK	CD164L2	CHEK1	EBI3	ICAM3	IL31RA	LILRA4	NFKBIL1	REL	TLR2	
BTLA	CD180	CHL1	EGF	ICAM4	IL32	LILRA5	NFRKB	RELA	TLR3	
C1QA	CD19	CHUK	EIF2AK2	ICOS	IL33	LILRA6	NKX2-3	RELB	TLR4	
C1QB	CD1A	CIITA	ELK1	IFI16	IL36A	LILRB1	NOD2	RFX1	TLR5	
C1QBP	CD1B	CISH	EMR3	IFI27	IL36G	LILRB2	NOL3	RFX5	TLR6	
C1QC	CD1C	CKLF	ENC1	IFI35	IL36RN	LILRB3	NOP9	RFXANK	TLR7	
C1QL1	CD1D	CLCF1	ENG	IFI44L	IL37	LILRB4	NOS2	RFXAP	TLR8	
C1QL2	CD1E	CLEC10A	ENTPD1	IFIH1	IL3RA	LILRB5	NPTN	RGS1	TLR9	
C1QL3	CD2	CLEC12A	EPO	IFIT1	IL4	LITAF	NRAS	RHAG	TLR9	
C1QL4	CD200R1	CLEC16A	EPX	IFIT1B	IL4I1	LPO	NT5E	RHCE	TMED7	

C1QTNF2	CD200R1L	CLEC4A	ERAP1	IFIT2	IL4R	LRP1	PADI4	RHD	TNF
C1QTNF3	CD207	CLEC4C	ERGIC2	IFIT3	IL5	LTA	PAFAH1B1	RIPK1	TNFAIP3
C1QTNF4	CD209	CLEC4D	ERLIN1	IFITM1	IL5RA	LTB	PAFAH1B2	RIPK2	TNFRSF10A
C1QTNF5	CD22	CLEC4E	ETS1	IFNA2	IL6	LTB4R	PAFAH1B3	RNASE7	TNFRSF10B
C1QTNF6	CD226	CLEC4M	ETS2	IFNAR2	IL6R	LTB4R2	PAFAH2	ROR1	TNFRSF10C
C1QTNF7	CD244	CLEC5A	F3	IFNB1	IL6ST	LTBR	PARP1	RORA	TNFRSF10D
C1R	CD247	CLEC6A	FADD	IFNG	IL7	LTF	PDCD1	RORC	TNFRSF11A
C1RL	CD248	CLEC7A	FAK	IFNGR1	IL7R	LY75	PDCD1LG2	RPA1	TNFRSF12A
C1S	CD27	CLECL1	FAS	IGF1R	IL8	LY86	PDGFB	S100A8	TNFRSF13B
C2	CD274	CLIP1	FASLG	IGF2R	IL9	LY9	PDGFRA	SAMSN1	TNFRSF13B
C3	CD276	CLIP2	FCAMR	IGJ	IL9R	LY96	PDGFRB	SARM1	TNFRSF13C
C3AR1	CD28	CLU	FCAR	IGLL1	ILF2	LYG2	PELI1	SCARB1	TNFRSF14
C4A	CD2AP	CMKLR1	FCER1A	IGSF8	ILF3	LYN	PELI2	SCARB2	TNFRSF17
C4B	CD2BP2	CMTM1	FCER1G	IKBKAP	INDO	LYZ	PF4	SCGB3A1	TNFRSF18
C4BPA	CD300A	CMTM2	FCER2	IKBKB	INSR	MAF	PGLYRP1	SDC1	TNFRSF1A
C4BPB	CD300C	CMTM3	FCGR1A	IKBKE	IRAK1	MAL	PGLYRP2	SDF2	TNFRSF1B
C5	CD300E	CMTM4	FCGR2A	IKBKG	IRAK1BP1	MAP2K3	PGLYRP3	SDF2L1	TNFRSF25
C5AR1	CD300LB	CMTM5	FCGR2B	IKZF1	IRAK2	MAP2K4	PGLYRP4	SELE	TNFRSF4
C6	CD300LF	CMTM6	FCGR2C	IKZF2	IRAK3	MAP2K6	PIAS3	SELL	TNFRSF8
C7	CD300LG	CMTM7	FCGR3A	IKZF3	IRAK4	MAP3K1	PIK3CG	SELP	TNFRSF9
C8A	CD302	CMTM8	FCGR3B	IL-17	IRF1	MAP3K14	PIK3R1	SELPLG	TNFSF10
C8B	CD320	COLEC12	FCGRT	IL10	IRF2	MAP4K4	PILRA	SEMA4D	TNFSF11
C8G	CD33	CR1	FCRL5	IL10RA	IRF4	MAPK1	PIM1	SEMA7A	TNFSF12
C9	CD34	CR1L	FCRLA	IL10RB	IRF5	MAPK10	PLA2G7	SERPING1	TNFSF13
C9orf47	CD36	CR2	FGFR1	IL11	IRF8	MAPK11	PLA2R1	SH2B3	TNFSF13B
CAMP	CD37	CRADD	FGFR2	IL11RA	IRF9	MAPK12	PLAA	SH2D1A	TNFSF14
CANX	CD38	CRLF1	FGFR3	IL12A	ISG20	MAPK13	PLAUR	SIGIRR	TNFSF15
CASP1	CD3D	CRLF2	FGFR4	IL12B	ITFG1	MAPK14	PLK3	SIGLEC1	TNFSF4
CASP10	CD3E	CRLF3	FGR	IL12B	ITGA1	MAPK3	PLXNC1	SIGLEC15	TNFSF8
CASP2	CD3EAP	CRP	FLT3	IL12RB1	ITGA2	MAPK6	PNP	SIGLEC5	TNIP1
CASP3	CD3G	CSF1	FOS	IL12RB2	ITGA2B	MAPK7	POMC	SIGLEC6	TOLLIP
CASP4	CD4	CSF1R	FOXK2	IL13	ITGA3	MAPK8	POU2AF1	SIRPA	TONSL
CASP6	CD40	CSF2	FOXN1	IL13RA1	ITGA4	MAPK8IP3	PPBP	SIVA1	TRADD
CASP7	CD40LG	CSF2RA	FOXO1	IL13RA2	ITGA5	MAPK9	PPIA	SLA	TRAF1
CASP8	CD44	CSF2RB	FOXO3	IL15	ITGA6	MAPKAPK2	PPP3CA	SLAMF1	TRAF2
CASP9	CD46	CSF3	FOXP3	IL15RA	ITGAD	MARCO	PPP3CB	SLAMF6	TRAF3
CCBP2	CD47	CSF3R	FRK	IL16	ITGAE	MASP1	PPP3CC	SLAMF7	TRAF3IP1
CCL1	CD48	CSN2	FUT3	IL17A	ITGAL	MASP2	PPP3R1	SLC3A2	TRAF4
CCL11	CD5	CTLA4	FYN	IL17B	ITGAM	MBL2	PPP3R2	SLC44A1	TRAF5
CCL13	CD52	CTSG	G6PD	IL17C	ITGAV	MBP	PRDM1	SLC4A1	TRAF6
CCL14	CD53	CTSS	GADD45A	IL17D	ITGAX	MBTPS1	PRDX6	SLC7A5	TRAF7
CCL15	CD58	CX3CL1	GNLY	IL17F	ITGB1	MCAM	PRF1	SMARCAL1	TSPYL2
CCL16	CD59	CX3CR1	GP1BA	IL17RA	ITGB2	MCL1	PRG2	SOCS1	TYK2
CCL17	CD5L	CXCL1	GP1BB	IL17RB	ITGB3	MDM2	PRKCD	SOCS2	ULBP1
CCL18	CD6	CXCL10	GP5	IL17RC	ITGB4	MFI2	PRKCQ	SOCS3	UNG
CCL19	CD63	CXCL11	GP9	IL17RD	JAK1	MICA	PRNP	SOCS4	WAS
CCL2	CD68	CXCL12	GPR183	IL17RE	JAK2	MICB	PROCR	SOCS5	WASF1
CCL20	CD69	CXCL13	GUSB	IL18	JAK3	MIF	PROM1	SOCS6	WASF3
CCL21	CD7	CXCL14	GYPA	IL18BP	JUN	MIF	PRSS16	SOCS7	VCAM1

CCL22	CD70	CXCL16	GYPB	IL18R1	KDR	MME	PSG1	SOD1	VCAN
CCL23	CD72	CXCL2	GYPC	IL18RAP	KEL	MMP9			VEGFA
CCL24	CD74	CXCL3	GYPE		KIF21B				WFDC12

Supplementary Table 4. Genes included in the immunogene panel sequencing. Gene names are presented as HUGO Gene Nomenclature Committee (HGNC) symbols.

	Mean target coverage	
Patient ID	CD4	CD8
1	385.3	413.2
2	406.8	440.9
3	404.4	405.8
4	202.9	197.8
5	204.1	343.5
6	193.6	365.6
7	134.4	100.3
8	454.7	441.7
9	397.0	386.2
10	434.2	208.0
11	242.2	141.4
12	163.9	228.4
13	436.6	460.0
14	475.5	421.3
15	419.6	505.9
16	404.0	355.4
17	707.5	366.0
18	351.5	343.9
19	309.1	396.0
20	109.5	127.9
21	469.9	569.8
22	632.3	458.1
23	304.9	1128.8
24	406.2	400.4
25	312.1	339.7
Median (IQR)	397.0 (223.2-435.4)	386.2 (284.1-441.3)
Mean (95% CI)	358.5 (298.6-418.4)	381.8 (300.7-462.9)
HC ID	CD4	CD8
HC1	1443.8	2501.1
HC2	1213.6	2345.6
HC3	1743.0	1165.6
HC4	1465.7	5839.6
HC5	1319.8	2161.6
HC6	1438.2	1383.0
HC7	722.9	1139.7
HC8	255.0	390.6
HC9	311.7	340.3
HC10	381.7	400.8
HC11	300.6	372.6
HC12	456.7	436.8
HC13	462.9	436.4
HC14	549.5	450.7
HC15	467.1	451.7
HC16	315.1	273.9
HC17	308.3	287.9
HC18	470.0	375.7
HC19	377.2	347.2
HC20	341.3	309.1
Median (IQR)	465.0 (321.7-1293)	436.6 (253.6-1329)
Mean (95% CI)	717.2 (481.9-952.5)	1070 (443.9-1697)

Supplementary Table 5. Sequencing coverage of immunogene-panel samples. Mean target coverage for samples sequenced with immunogene-panel sequencing are presented in the table. The median sequencing depth for heathy controls was higher than for RA patients. Abbreviations: IQR, interquartile range; CI, confidence interval.

Exome sequencing

Pat.	Cell fraction	chr	pos (hg 19)	ref	var	Gene	Description	Effect	Exon	Transcript (Ensembl 66)	AA change	CD4+ ref reads	CD4+ var reads	CD4+var freq	Vβ+ ref reads	Vβ+ var reads	Vβ+ var freq	somatic p-value
1	CD8+Vβ13.1+	12	15677849	A	T	*PTPRO*	protein tyrosine phosphatase, receptor type, O	NS	11	ENST00000281171	M665L	92	0	0	46	22	32	6.613E-10
1	CD8+Vβ13.1+	11	88386546	C	T	*GRM5*	glutamate receptor, metabotropic 5	NS	4	ENST00000418177	V313M	76	0	0	48	13	21	0.000012285
1	CD8+Vβ13.1+	4	16017836	T	A	*PROM1*	prominin 1	NS	9	ENST00000447510	N344Y	44	0	0	36	14	28	0.000053814
1	CD8+Vβ13.1+	4	100572576	C	T	*RP11-766F14.2.*1	uncharacterized protein	NS	1	ENST00000511828	R1077H	33	0	0	20	11	35	0.00011387
1	CD8+Vβ13.1+	21	10908835	C	G	*TPTE*	transmembrane phosphatase with tensin homology	NS	23	ENST00000361285	E504Q	117	0	0	97	11	10	0.00023594
1	CD8+Vβ13.1+	9	123722610	A	T	*C5*	complement component 5	NS	38	ENST00000223642	C1532S	105	0	0	93	11	11	0.0003501
1	CD8+Vβ13.1+	22	25627599	C	T	*CRYBB2*	crystallin, beta B2	NS	6	ENST00000398215	R160C	49	0	0	41	9	18	0.0014474
1	CD8+Vβ13.1+	1	205038666	G	A	*CNTN2*	contactin 2 (axonal)	NS	17	ENST00000331830	G725R	25	0	0	18	7	28	0.0048126
1	CD8+Vβ13.1+	20	16360582	C	T	*KIF16B*	kinesin family member 16B	NS	19	ENST00000408042	E689K	82	0	0	68	7	9	0.0048726
1	CD8+Vβ13.1+	5	133496777	A	AG	*SKP1*	S-phase kinase-associated protein 1	FS	5	ENST00000517625	E73fs	33	0	0	15	5	25	0.0054027
1	CD8+Vβ13.1+	17	37627581	G	A	*CDK12*	cyclin-dependent kinase 12	NS	2	ENST00000447079	G499E	58	0	0	40	6	13	0.006173
1	CD8+Vβ13.1+	2	216809684	A	T	*MREG*	melanoregulin	NS	5	ENST00000236976	F183I	47	0	0	32	6	16	0.0063122
3	CD8+Vβ7.2+	1	167805547	C	A	*ADCY10*	adenylate cyclase 10 (soluble)	NS	23	ENST00000367851	M1103I	154	0	0	85	28	25	3.9222E-12
3	CD8+Vβ7.2+	4	155468910	T	C	*PLRG1*	pleiotropic regulator 1	NS	3	ENST00000393905	Q71R	109	0	0	90	7	7	0.0045591
3	CD8+Vβ7.2+	4	95201870	T	G	*SMARCAD1*	SWI/SNF-related, matrix-associated actin-dependent regulator of chromatin, subfamily a, containing DEAD/H box 1	NS	20	ENST00000359052	L851W	50	0	0	54	8	13	0.0071108
4	CD8+V1+	21	33044259	T	C	*SCAF4*	SR-related CTD-associated factor 4	NS	20	ENST00000286835	Q966R	51	0	0	30	14	32	5.6241E-06
4	CD8+Vβ1+	1	220160577	T	C	*EPRS*	glutamyl-prolyl-tRNA synthetase	NS	20	ENST00000366923	N982S	82	0	0	59	14	19	0.000012775
4	CD8+Vβ1+	4	15717329	GT	G	*BST1*	bone marrow stromal cell antigen 1	FS	7	ENST00000382346	F220fs	39	0	0	25	11	31	0.00012266
4	CD8+Vβ1+	1	36204776	C	T	*CLSPN*	claspin	NS	20	ENST00000318121	G1131S	47	0	0	28	8	22	0.00076719
4	CD8+Vβ1+	15	81614763	G	A	*STARD5*	StAR-related lipid transfer (START) domain containing 5	STOP	3	ENST00000302824	Q90*	35	0	0	21	8	28	0.00096972
4	CD8+Vβ1+	3	44285882	TA	T	*TOPAZ1*	testis and ovary specific PAZ domain containing T	FS	2	ENST00000309765	N628fs	71	1	1.39	65	11	14	0.0030399
4	CD8+Vβ1+	19	12975742	C	T	*MAST1*	microtubule associated serine/threonine kinase 1	NS	13	ENST00000251472	R496C	35	0	0	19	6	24	0.0035375
4	CD8+Vβ1+	16	5115776	C	T	*C16orf89*	chromosome 16 open reading frame 89	NS	1	ENST00000350219	R83K	36	0	0	33	8	20	0.0045408
4	CD8+Vβ1+	6	4892329	C	G	*CDYL*	chromodomain protein, Y-like	NS	4	ENST00000328908	A190G	94	0	0	80	7	8	0.0052041

Immunogene panel sequencing

Pat.	Cell fraction	chr.	pos (hg 19)	ref	var	Gene	Description	Effect	Exon	Transcript (Ensembl 66)	AA change	CD4+ ref reads	CD4+ var reads	CD4+ var freq	CD8+ ref reads	CD8+ var reads	CD8+ var freq	somatic p-value
1	CD8+	4	16017836	T	A	*PROM1*	prominin 1	NS	4	ENST00000447510	N344Y	713	0	0	572	88	13	4.0181E-30
1	CD8+	1	17674463	G	A	*PADI4*	peptidyl arginine deiminase, type IV	NS	10	ENST00000375448	A359T	236	1	0.42	217	46	17	4.7195E-13
1	CD8+	9	123722610	A	T	*C5*	complement component 5	NS	9	ENST00000223642	C1532S	612	0	0	540	38	7	6.335E-13
1	CD8+	7	73803421	*	G	*CLIP2*	CAP-GLY domain containing linker protein 2	SSA		ENST00000223398		176	0	0	191	11	5	0.00089102
2	CD8+	5	131821384	C	T	*IRF1*	interferon regulatory factor 1	NS	8	ENST00000245414	G231E	645	0	0	817	57	7	9.3049E-15
2	CD8+	22	30866019	C	T	*SEC14L3*	SEC14-like 3 (S. Cerevisiae)	STOP	4	ENST00000215812	W74*	629	0	0	669	46	6	1.2234E-13
2	CD8+	1	160460409	A	C	*SLAMF6*	SLAM family member 6	NS	4	ENST00000368057	F238C	579	0	0	584	31	5	8.0288E-10

4	CD8+	4	157 173 29	G T	G	*BST1*	bone marrow stromal cell antigen 1	FS	7	ENST000003 82346	F220fs	217	0	0	222	20	8	1.870 3E-06
5	CD8+	17	697 875 8	C	T	*CLEC10A*	C-type lectin domain family 10, member A	NS	8	ENST000002 54868	A235T	189	0	0	263	30	10	1.767 E-07
HC 5	CD8+	8	274 722 22	T	C	*CLU*	clusterin	NS	1	ENST000005 60366	H26R	462	0	0	576	93	14	3.691 8E-23

Supplementary Table 6. Somatic mutations discovered in patients and controls. Exome sequencing of 3 patients' CD8+Vb+ clones (size 40-55% of CD8+) revealed 24 somatic mutations in the expanded clone. CD4+ cells were used as a germline control. Immunogene panel sequencing of 23 patients' and 7 healthy controls' CD4+ and CD8+ cells revealed 10 somatic mutations in CD8+. PROM1, C5, BST1 mutations were discovered with both methods. In all cases, the mutations were validated with amplicon sequencing. *) GGCTGACCCCAGC Abbreviations: Pat., patient; chr, chromosome; Pos, postion; ref, reference allele; var, variant allele; NS, non-synonymous coding mutation; FS, frameshift; STOP, nonsense mutation; SSA, splice site acceptor; AA, amino acid; freq, frequency.

Gene	Mutation identified in the patient	Genomic coordinates (hg19)	No. of samples with the same mutation in COSMIC	Amino-acid change in COSMIC	Tissue	Genomic coordinates in COSMIC (hg19)	COSMIC ID
CRYBB2	R160C	22:g.25627599C>T	1	R160C	Central nervous system	22:g.25627599C>T	COSM3405558
IRF1	G231E	5:g.131821384C>T	1	G231E	NS	5:g.131821384G>A	COSM5867257
CLEC10A	A235T	17:g.6978758C>T	1	A235T	Endometrium	7:g.6978758G>A	COSM983742
SMARCAD1	L851W	4:g.95201870T>G	1	L851W	breast	4:g.95201870T>G[1]	COSM213551
PTPRO	M665L	12:g.15677849A>T	1	M665I	endometrium	12:g.15677851G>A	COSM937606
CDYL	A190G	6:g.4892329C>G	1	A190V	large intestine	6:g.4892329C>T	COSM3353854
SLAMF6	F238C	1:g.160460409A>C	1	F238F	Stomach	1:g.160460408C>T	COSM4024184

Supplementary Table 7. Somatic mutations previously reported in the COSMIC database. All identified somatic mutations were queried against the Catalogue of somatic mutations in cancer (COSMIC; http://cancer.sanger.ac.uk/cosmic)[2] database. Four exactly same mutations occurred in the RA patients as well as in COSMIC. COSMIC also reported three mutations that affect the same amino acid than the mutation in found in our study, but the amino-acid change was different. [1]The protein amino-acid change in COSMIC was L849W but for the canonical transcript the change is L851W.

Patient ID	Clone size (% of CD8+)	AA sequence	nucleotide sequence	TCRBV gene	TCRBJ gene	TCRBD gene	Target antigen	Reference no.
19	3.1	CASSSANY GYTF	CTGAAGATCCAGCCCTC AGAACCCAGGGACTCAG CTGTGTACTTCTGTGCC AGCAGTTCAGCTAACTA TGGCTACACCTTCGGTT CG	12-3*01 or 12-4*01	01-02*01	01-01*01	PP65	14, 16, and 17
22	3.7	CASSSANY GYTF	CTGAAGATCCAGCCCTC AGAACCCAGGGACTCAG CTGTGTACTTCTGTGCC AGCAGTTCCGCTAACTA TGGCTACACCTTCGGTT CG	12-3*01 or 12-4*01	01-02*01	02-01	PP65	14, 16, and 17
79	1.1	CASSSANY GYTF	CTGAAGATCCAGCCCTC AGAACCCAGGGACTCAG CTGTGTACTTCTGTGCC AGCAGTTCGGCTAACTA TGGCTACACCTTCGGTT CG	12-3*01 or 12-4*01	01-02*01	02-01	PP65	14, 16, and 17
HC4	1.3	CASSLGQ AYEQYF	AAGATCCAGCGCACACA GCAGGAGGACTCCGCCG TGTATCTCTGTGCCAGC AGCTTAGGCCAGGCCTA CGAGCAGTACTTCGGGC CG	07-08*01	02-07*01	01-01*01	EBV	17 and 27

Supplementary Table 8. Public and shared TCR sequences detected in CD8+ T-cell clones exceeding 1% of all CD8+. We focused our studies on major CD8+ T-cell clones. We compared the TCRB sequences from patients and healthy controls to previously published results: virus-specific TCRs[3-18], and sequences observed previously in RA[19-33]. Three patients harbored a major clone with a public TCRB sequence, which was directed against CMV. One of the healthy controls harbored a clone targeting EBV[18], but the same TCRB sequence has been reported as well in RA synovia[28].

Assay	F primer	R primer
ADCY10	TTCAGTGGAAAGGGGTGCTG	CCCTCTGGAATCTTGCCAGT
BST1	TGACCTGGTGATCAGAGCCA	GGGTCCCCCAATTTCATGCA
C16orf89	CCTGGCCTCCTCTCTCCTTA	TTACTGACAGCACTGCCACC
C5	TGGGCTCATGAGAAACCGT	AGGGGATCACATGGAATGTTGT
CDK12	AAGGAGTCCAAGGGTTCACC	TAGTTGGTAGAGGGGGTGGG
CDYL	GGACCTCTCCCAACAATGCT	GGTCCAGTTTCTCAGGGCTC
CLEC10A	CCTGGCTTCCAGTTCCTGAG	AAGTCACCACTGCCCTTCTG
CLIP2	ATGCAGTGGGGTATGTCACC	CTCCTTCCGCAGCAGCTC
CLSPN	TTCAGGCTGGTCCTGAACTC	GATGAGGAACTGCAGAGTCAAA
CLU	CGAGCTGTGTCATCCCTCTC	TGGAGCCAGCACAGCTATTC
CNTN2	GGTTCCCATCTTACGGTGCT	TGAGTCAGAGCTGTGCAGTG
CRYBB2	AATGGTTGGGAGGCTTCACC	TCACTCTCTGGGAGGTCTGG
EPRS	TCTGTTTCTTAGGCCCCTGC	GTGTGCTTAGACAATGACTTGAGT
GRM5	AACCAAGGGTTTCGGTGGTT	GGCCCAAAATTTCCATGGTGT
IRF1	CGTGAATGTGGCACTTGTGG	GAACTGGAACTGCAGGGTGA
KIF16B	TTTTCCTTTTGGAGCTGGTC	AAGGAGACAGAAATCGTGCAG
MAST1	GGTGCTATGGTGCATGTCCA	TGGGGATCTCAGGGATGAGG
MREG	TGACTGCTGAGTCCTCATGG	GGGTGGGTTTTGTTGTTGTT
PADI4	GCAAGGGTGAGAGTGAGTGG	GGCAGGCTAAAAGGAGGGAG
PLRG1	CCATGAGGATCAAAACTTCCA	TTTCTACGTGAATGGGCAAA
PROM1	GGCAACCTTTCTCTAGAATTTCA	TCATGAGGAATTGTTTTGAAGG
PTPRO	TGGGATTTCATGCTCTGCTG	ACAGACACACAATGCTGGCA
RP11-766F14.2.1	CCTGCTCTGGGGTAACACTG	CAGCCGAGCTCAGACTTCAA
SCAF4	CAAGTCTCTGTGCCTGTCCC	GGGGGTCCTGAAGACAGAGA
SEC14L3	AACATCTTAACCTAGGCCTGGAC	TCCGCAAGGTGAGACCTATC
SKP1	AGTTCAAAAAGTGTTCCTTGGTCA	TGGCTCAAGTCAACCTCTAGC
SLAMF6	CTCCCTCCTCCTGTCTTCCA	CATAGGACTCCCACCCCAGA
SMARCAD1	GGAACCTACACATTGTGAGGC	AGCAAGTCAGTGTGCCAGAA
STARD5	GTTTGGGGCTTCTCTCCCTC	TGATTGATGCGTTGGGGTCA
TOPAZ1	AGAGGAACTGAGCAGAAGAGG	TCAGCCGTCCTGTGTTCAAT
TPTE	GGAACAATGGTGGTTCAAGG	GGAACTTCAATTTCTTTGGAATG

Supplementary Table 9. Amplicon sequencing primers.

Supplementary References

Virus-specific TCR sequences[3-18]

TCR sequences reported in RA patients[19-33]

1 Reveille, JD. The genetic contribution to the pathogenesis of rheumatoid arthritis. *Curr Opin Rheumatol.* 1998;**10**(3): 187-200.

2 Forbes, SA, Beare, D, Gunasekaran, P *et al.* COSMIC: exploring the world's knowledge of somatic mutations in human cancer. *Nucleic Acids Res.* 2014.

3 Argaet, VP, Schmidt, CW, Burrows, SR *et al.* Dominant selection of an invariant T cell antigen receptor in response to persistent infection by Epstein-Barr virus. *J Exp Med.* 1994;**180**(6): 2335-2340.

4 Babel, N, Brestrich, G, Gondek, LP *et al.* Clonotype analysis of cytomegalovirus-specific cytotoxic T lymphocytes. *J Am Soc Nephrol.* 2009;**20**(2): 344-352.

5 Cameron, TO, Cohen, GB, Islam, SA & Stern, LJ. Examination of the highly diverse CD4(+) T-cell repertoire directed against an influenza peptide: a step towards TCR proteomics. *Immunogenetics.* 2002;**54**(9): 611-620.

6 Hamel, Y, Rohrlich, P, Baron, V *et al.* Characterization of antigen-specific repertoire diversity following in vitro restimulation by a recombinant adenovirus expressing human cytomegalovirus pp65. *Eur J Immunol.* 2003;**33**(3): 760-768.

7 Hennecke, J, Carfi, A & Wiley, DC. Structure of a covalently stabilized complex of a human alphabeta T-cell receptor, influenza HA peptide and MHC class II molecule, HLA-DR1. *EMBO J.* 2000;**19**(21): 5611-5624.

8 Jones, CM, Lake, RA, Lamb, JR & Faith, A. Degeneracy of T cell receptor recognition of an influenza virus hemagglutinin epitope restricted by HLA-DQ and -DR class II molecules. *Eur J Immunol.* 1994;**24**(5): 1137-1142.

9 Klinger, M, Pepin, F, Wilkins, J *et al.* Multiplex Identification of Antigen-Specific T Cell Receptors Using a Combination of Immune Assays and Immune Receptor Sequencing. *PLoS One.* 2015;**10**(10): e0141561.

10 Lim, A, Trautmann, L, Peyrat, MA *et al.* Frequent contribution of T cell clonotypes with public TCR features to the chronic response against a dominant EBV-derived epitope: application to direct detection of their molecular imprint on the human peripheral T cell repertoire. *J Immunol.* 2000;**165**(4): 2001-2011.

11 Prevost-Blondel, A, Chassin, D, Zeliszewski, D *et al.* Preferential usage of the T-cell receptor by influenza virus hemagglutinin-specific human CD4+ T lymphocytes: in vitro life span of clonotypic T cells. *J Virol.* 1995;**69**(12): 8046-8050.

12 Price, DA, Brenchley, JM, Ruff, LE *et al.* Avidity for antigen shapes clonal dominance in CD8+ T cell populations specific for persistent DNA viruses. *J Exp Med.* 2005;**202**(10): 1349-1361.

13 Retiere, C, Prod'homme, V, Imbert-Marcille, BM, Bonneville, M, Vie, H & Hallet, MM. Generation of cytomegalovirus-specific human T-lymphocyte clones by using autologous B-lymphoblastoid cells with stable expression

of pp65 or IE1 proteins: a tool to study the fine specificity of the antiviral response. *J Virol.* 2000;**74**(9): 3948-3952.
14 Schwanninger, A, Weinberger, B, Weiskopf, D *et al.* Age-related appearance of a CMV-specific high-avidity CD8+ T cell clonotype which does not occur in young adults. *Immun Ageing.* 2008;**5**(14.
15 Trautmann, L, Rimbert, M, Echasserieau, K *et al.* Selection of T cell clones expressing high-affinity public TCRs within Human cytomegalovirus-specific CD8 T cell responses. *J Immunol.* 2005;**175**(9): 6123-6132.
16 van Leeuwen, EM, Remmerswaal, EB, Heemskerk, MH, ten Berge, IJ & van Lier, RA. Strong selection of virus-specific cytotoxic CD4+ T-cell clones during primary human cytomegalovirus infection. *Blood.* 2006;**108**(9): 3121-3127.
17 Weekes, MP, Wills, MR, Mynard, K, Carmichael, AJ & Sissons, JG. The memory cytotoxic T-lymphocyte (CTL) response to human cytomegalovirus infection contains individual peptide-specific CTL clones that have undergone extensive expansion in vivo. *J Virol.* 1999;**73**(3): 2099-2108.
18 Zvyagin, IV, Pogorelyy, MV, Ivanova, ME *et al.* Distinctive properties of identical twins' TCR repertoires revealed by high-throughput sequencing. *Proc Natl Acad Sci U S A.* 2014;**111**(16): 5980-5985.
19 Alam, A, Lambert, N, Lule, J *et al.* Persistence of dominant T cell clones in synovial tissues during rheumatoid arthritis. *J Immunol.* 1996;**156**(9): 3480-3485.
20 Davey, MP, Burgoine, GA & Woody, CN. TCRB clonotypes are present in CD4+ T cell populations prepared directly from rheumatoid synovium. *Hum Immunol.* 1997;**55**(1): 11-21.
21 Fazou, C, Yang, H, McMichael, AJ & Callan, MF. Epitope specificity of clonally expanded populations of CD8+ T cells found within the joints of patients with inflammatory arthritis. *Arthritis Rheum.* 2001;**44**(9): 2038-2045.
22 Gonzalez-Quintial, R, Baccala, R, Pope, RM & Theofilopoulos, AN. Identification of clonally expanded T cells in rheumatoid arthritis using a sequence enrichment nuclease assay. *J Clin Invest.* 1996;**97**(5): 1335-1343.
23 Goronzy, JJ, Bartz-Bazzanella, P, Hu, W, Jendro, MC, Walser-Kuntz, DR & Weyand, CM. Dominant clonotypes in the repertoire of peripheral CD4+ T cells in rheumatoid arthritis. *J Clin Invest.* 1994;**94**(5): 2068-2076.
24 Grom, AA, Thompson, SD, Luyrink, L, Passo, M, Choi, E & Glass, DN. Dominant T-cell-receptor beta chain variable region V beta 14+ clones in juvenile rheumatoid arthritis. *Proc Natl Acad Sci U S A.* 1993;**90**(23): 11104-11108.
25 Howell, MD, Diveley, JP, Lundeen, KA *et al.* Limited T-cell receptor beta-chain heterogeneity among interleukin 2 receptor-positive synovial T cells suggests a role for superantigen in rheumatoid arthritis. *Proc Natl Acad Sci U S A.* 1991;**88**(23): 10921-10925.
26 Ikeda, Y, Masuko, K, Nakai, Y *et al.* High frequencies of identical T cell clonotypes in synovial tissues of rheumatoid arthritis patients suggest the occurrence of common antigen-driven immune responses. *Arthritis Rheum.* 1996;**39**(3): 446-453.

27 Khazaei, HA, Lunardi, C & So, AK. CD4 T cells in the rheumatoid joint are oligoclonally activated and change during the course of disease. *Ann Rheum Dis.* 1995;**54**(4): 314-317.
28 Klarenbeek, PL, de Hair, MJ, Doorenspleet, ME *et al.* Inflamed target tissue provides a specific niche for highly expanded T-cell clones in early human autoimmune disease. *Ann Rheum Dis.* 2012;**71**(6): 1088-1093.
29 Li, Y, Sun, GR, Tumang, JR, Crow, MK & Friedman, SM. CDR3 sequence motifs shared by oligoclonal rheumatoid arthritis synovial T cells. Evidence for an antigen-driven response. *J Clin Invest.* 1994;**94**(6): 2525-2531.
30 Lunardi, C, Marguerie, C & So, AK. An altered repertoire of T cell receptor V gene expression by rheumatoid synovial fluid T lymphocytes. *Clin Exp Immunol.* 1992;**90**(3): 440-446.
31 Paliard, X, West, SG, Lafferty, JA *et al.* Evidence for the effects of a superantigen in rheumatoid arthritis. *Science.* 1991;**253**(5017): 325-329.
32 Struyk, L, Hawes, GE, Dolhain, RJ *et al.* Evidence for selective in vivo expansion of synovial tissue-infiltrating CD4+CD45RO+ T-lymphocytes on the basis of CDR3 diversity. *Ann N Y Acad Sci.* 1995;**756**(204-207.
33 Sun, W, Nie, H, Li, N *et al.* Skewed T-cell receptor BV14 and BV16 expression and shared CDR3 sequence and common sequence motifs in synovial T cells of rheumatoid arthritis. *Genes Immun.* 2005;**6**(3): 248-261.
